# Predicting Suicidal Ideation, Planning, and Attempts among the Adolescent Population of the United States

**DOI:** 10.3390/healthcare12131262

**Published:** 2024-06-25

**Authors:** Hamed Khosravi, Imtiaz Ahmed, Avishek Choudhury

**Affiliations:** Industrial and Management Systems Engineering, West Virginia University, Morgantown, WV 26506, USAimtiaz.ahmed@mail.wvu.edu (I.A.)

**Keywords:** depression, suicide, student mental health, public health

## Abstract

Suicide is the second leading cause of death among individuals aged 5 to 24 in the United States (US). However, the precursors to suicide often do not surface, making suicide prevention challenging. This study aims to develop a machine learning model for predicting suicide ideation (SI), suicide planning (SP), and suicide attempts (SA) among adolescents in the US during the coronavirus pandemic. We used the 2021 Adolescent Behaviors and Experiences Survey Data. Class imbalance was addressed using the proposed data augmentation method tailored for binary variables, Modified Synthetic Minority Over-Sampling Technique. Five different ML models were trained and compared. SHapley Additive exPlanations analysis was conducted for explainability. The Logistic Regression model, identified as the most effective, showed superior performance across all targets, achieving high scores in recall: 0.82, accuracy: 0.80, and area under the Receiver Operating Characteristic curve: 0.88. Variables such as sad feelings, hopelessness, sexual behavior, and being overweight were noted as the most important predictors. Our model holds promise in helping health policymakers design effective public health interventions. By identifying vulnerable sub-groups within regions, our model can guide the implementation of tailored interventions that facilitate early identification and referral to medical treatment.

## 1. Introduction

Suicide, often a consequence of chronic mental distress, represents a serious health concern accounting for roughly 1.4% of global deaths [1,2,3]. It is the second leading cause of death among individuals aged 5 to 24 in the United States (US) [4,5]. The 2019 coronavirus pandemic further exacerbated this issue [6], particularly among the adolescent population [7]. It is important to recognize that suicide is not an abrupt decision. The progression towards suicide begins with ideation, evolves through planning, and ultimately culminates in an attempt. However, the precursors to suicide typically do not surface, making suicide prevention challenging. Timely recognition of these precursors is crucial for suicide prevention.

Machine learning (ML) is a powerful tool that has been increasingly used to help predict cases of suicidal thoughts, attempts, and deaths. This is a significant step forward in the effort to prevent suicide, as highlighted by research from several experts in the field [8,9,10,11]. According to a recent review [12], various machine learning techniques, such as Decision Trees (DT), Support Vector Machines (SVM), Logistic Regression (LR), K-Nearest Neighbors (KNN), Random Forests (RF), and Naïve Bayes (NB) have been utilized in this research. Despite the promising results, some studies have raised concerns about the overestimation of the effectiveness of certain methods, like Random Forests, in predicting suicide risks [13]. The data used to train these machine learning models come from diverse sources. Social media has been a notable source [14,15,16,17,18], as it provides real-time and authentic user expressions, which can be critical for predicting suicidal behavior. Other important data sources include psychological measures and health conditions obtained through questionnaires and interviews [19,20,21,22,23], changes in brain activity observed through fMRI scans [24,25], patient information from electronic health records [26,27], responses from the Brief Symptom Rating Scale, and the Suicidal Ideation Questionnaire [28,29]. While models trained on clinical data have proven to be effective, their use is mostly confined to healthcare professionals. This limitation arises because the public does not have access to such specialized data, making it challenging for non-professionals to leverage these models to identify and prevent suicide risks among their friends and family. This gap underscores the need for models that can operate effectively with more widely accessible data, thereby extending the reach of these life-saving technologies to the broader community.

In this study we contribute to the field of suicide prevention by predicting suicide ideation, planning, and attempts among young adults in the US during the coronavirus pandemic. Additionally, we propose a novel method for data augmentation—a modified Synthetic Minority Over-sampling Technique (SMOTE) tailored for binary features—to address the concern of imbalanced datasets.

## 2. Methods and Materials

All data used in this study are deidentified public health surveillance data and, therefore, not subject to institutional review board approval. We used the 2021 Adolescent Behaviors and Experiences Survey (ABES) [30]. The survey was conducted during the spring 2021 academic semester, spanning from January to June 2021. A total of 7998 students from 128 public and private schools participated in the survey. After processing the responses, valid data were obtained from 7705 questionnaires. The survey gathered data from a representative sample of students from the 9th to 12th grades in the United States during the COVID-19 pandemic. The survey consists of 110 questions encompassing a range of topics, including emotional well-being, racism, violence and unintentional injuries, sexual behaviors, substance use, dietary habits, and physical activity.

**Figure 1:**
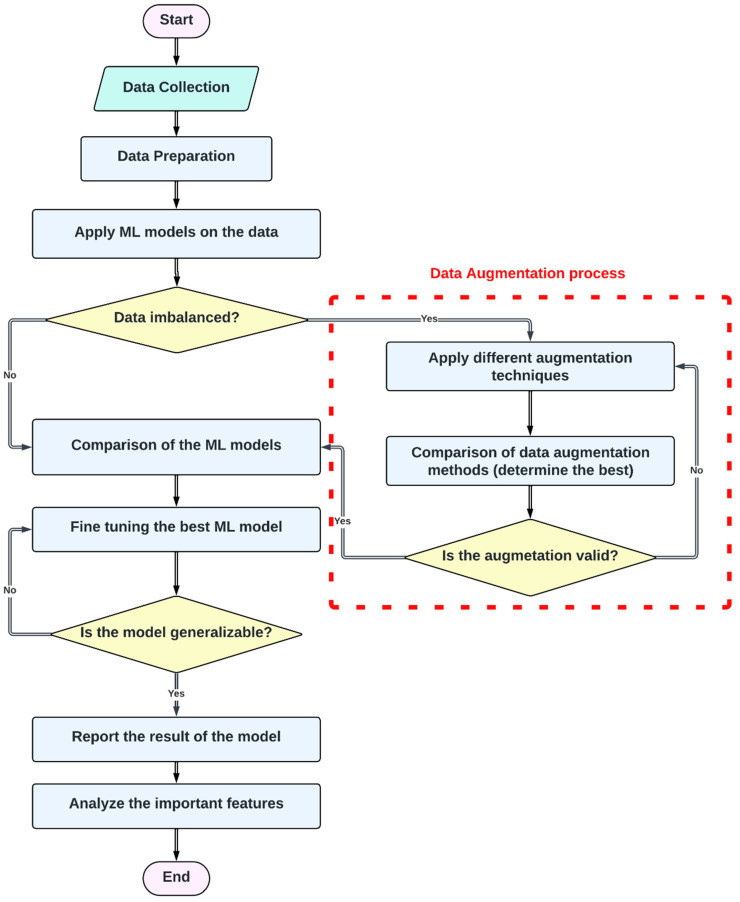
The flowchart of the methodology applied in the study.

Figure 1 shows the overall process of the methodology utilized in this study.

For data preparation, we employed various techniques for handling missing values, which demonstrated effectiveness in prior studies [31,32,33]. These techniques included median and mode imputation, random imputation based on distribution, group-wise imputation, clustering, and Multiple Imputation by Chained Equations (MICE). Categorical features were transformed into dummy variables to ensure compatibility with the models. Highly correlated (<0.75) variables were also removed.

The final dataset, optimized for modeling, comprised 6345 participants. Three questions within the dataset were designated as the target (class) variables of interest: suicide ideation, suicide planning, and suicide attempt. Class 1 indicates the event happening. [see Supplementary Materials Section S1 for more information about the data preparation].

- *Suicide Ideation* (*SI*)—The variable was measured using the following question: During the past 12 months, did you ever seriously consider attempting suicide? The responses were recorded as ‘Yes’ or ‘No’. In our model, ‘Yes’ was coded as 1;
- *Suicide planning* (*SP*)—The variable was measured using the following question: During the past 12 months, did you make a plan about how you would attempt suicide? The responses were recorded as ‘Yes’ or ‘No’;
- *Suicide Attempt* (*SA*)—The variable was measured using the following question: During the past 12 months, how many times did you actually attempt suicide? The responses were recorded as 0 times, 1 time, 2 or 3 times, 4 or 5 times, or 6 or more times. In our study, we coded all responses indicating at least one attempt as 1 and the remainder as 0.

Subsequently, we applied five ML models to the datasets, each targeting the variables of suicide ideation, suicide planning, and suicide attempt. The models selected were Decision Tree (DT), Random Forest (RF), Support Vector Machines (SVM), Logistic Regression (LR), and eXtreme Gradient Boosting (XGB). The rationale behind choosing these specific models lies in their demonstrated efficacy in predicting binary targets [34,35,36].

After the initial application of the machine learning models, we assessed the dataset balance for each target variable. Given the prevalence of imbalanced data, we employed our specially modified Synthetic Minority Over-sampling Technique (SMOTE), tailored for binary features. We identified the binary columns within the dataset. Following the resampling under the standard SMOTE framework, we applied a 0.50 threshold to the resampled data for binary features, converting values above this threshold to 1 and the rest to 0. This step guarantees the preservation of the binary characteristics of specific features, ensuring that the binary nature of certain features is preserved during the data augmentation process. Additionally, we used a few other common augmentation techniques such as the standard SMOTE [37], Gaussian Copula [38], and Conditional Tabular Generative Adversarial Network (CTGAN) [39].

To assess the efficacy of the data augmentation techniques, ML models were applied to the balanced datasets. The superior techniques were selected based on their ability to enhance the recall scores. Emphasis was placed on the recall of the minor class over accuracy, as it better indicates the models’ precision in predicting suicide ideation, planning, and attempts. An analysis of augmentation validity was followed, ensuring the synthetic data mirrored the real data’s distribution and structure.

The most effective ML model was then identified, and it underwent additional fine-tuning for each target variable. The fine-tuning process involved hyperparameter tuning, application of varying weights to the classes, and feature selection with Recursive Feature Elimination (RFE). To confirm the model’s accuracy, robustness, and generalizability to new data, the ROC analysis was performed on the best models. Finally, SHapley Additive exPlanations (SHAP) analysis was conducted to evaluate the key features that significantly influenced the prediction of each target.

## 3. Result

Table 1 shows the participant characteristics and distribution of suicide ideation, planning, and attempts across various demographic segments.

Table 2 shows the model comparison of 5 classifiers before data augmentation. The ML models consistently presented high accuracy, with LR having the best recall score for the target class. However, we noted a decline in sensitivity from predicting suicide ideation to attempts, underscoring the need for methodological improvements like data augmentation to address dataset imbalances in suicidal behaviors.

**Figure 2:**
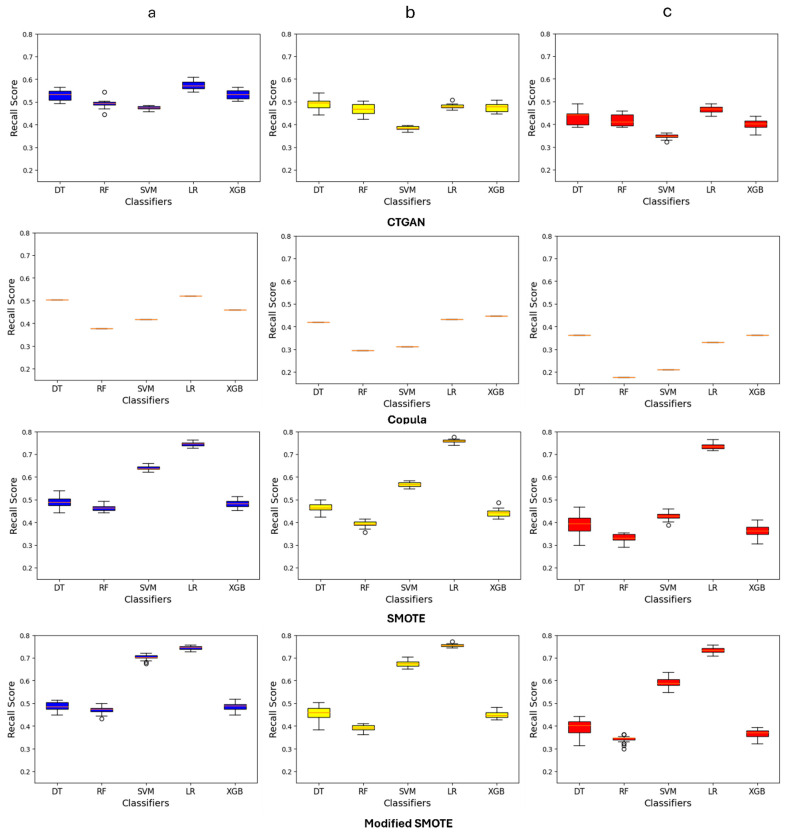
The overall recall score of different ML models after applying different augmentation techniques on target features: (**a**) suicide ideation; (**b**) suicide planning; and (**c**) suicide attempt.

Data augmentation techniques were employed to generate synthetic data for the minor class. To show the consistency and robustness of the methods, each of the techniques was applied 25 times, and the result is shown in Figure 2 [see Supplementary Materials Section S2 for additional analyses]. The figure shows that both SMOTE and the modified SMOTE techniques outperformed other data augmentation methods in improving recall scores for Class 1 across all targets. The modified SMOTE, particularly with the SVM model, showed a significant enhancement in recall compared to the standard SMOTE. For suicide ideation, planning, and attempt, the SVM with the Modified SMOTE technique attained recall scores of 0.71, 0.68, and 0.59, respectively, marking a notable enhancement over the standard SMOTE, which scored 0.64, 0.57, and 0.44 for these targets. The most impressive results were observed with the LR model, which achieved the highest median recall scores. Specifically, for the targets of suicide ideation, planning, and attempt, the recall scores were 0.74, 0.73, and 0.76, respectively.

**Figure 3:**
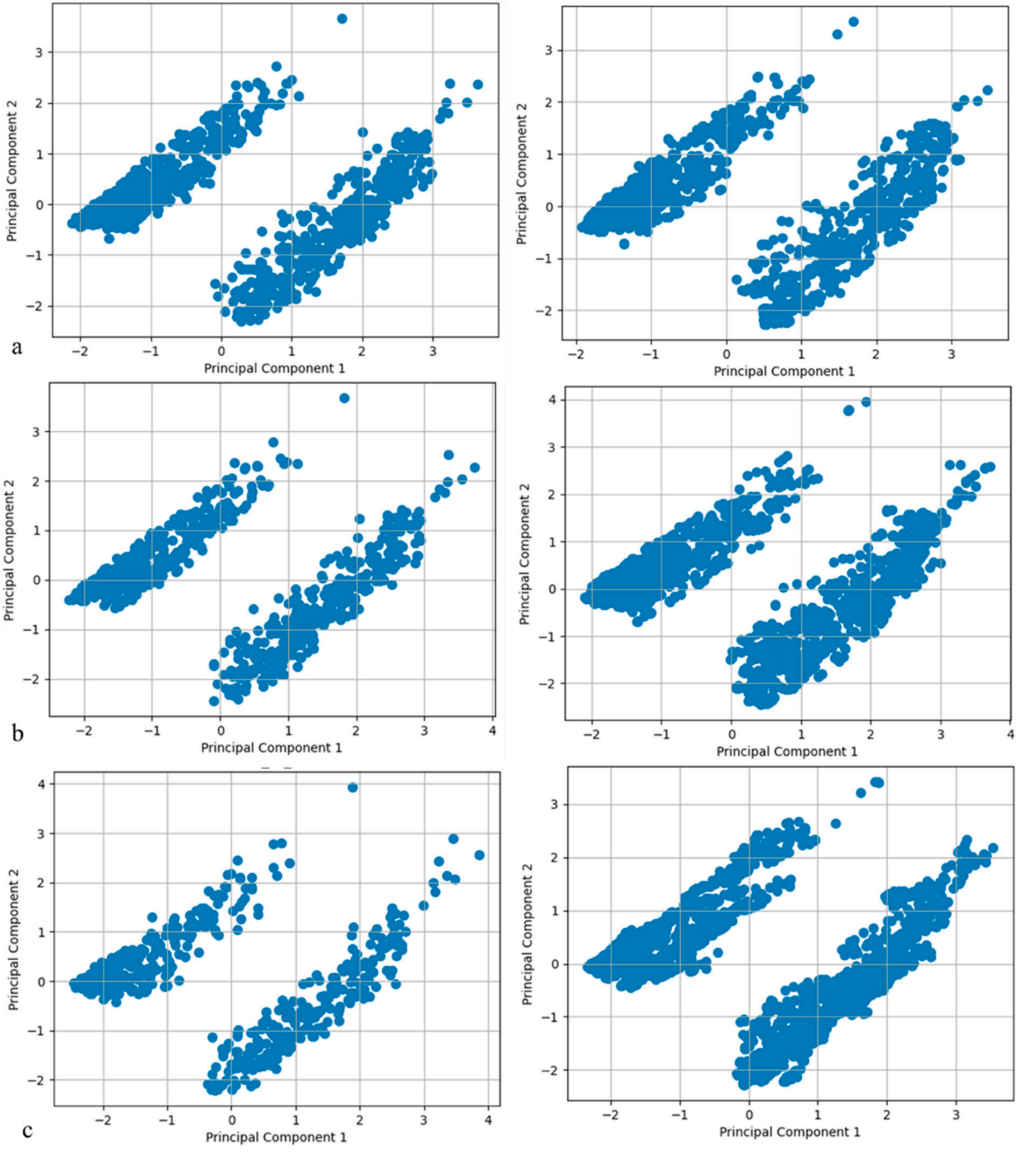
Comparison between the real ((**left**) side) and synthetic data ((**right**) side) considering the first two components of PCA: (**a**) suicide ideation; (**b**) suicide planning; and (**c**) suicide attempt.

Next, the Modified SMOTE technique’s effectiveness in generating synthetic data was evaluated using a Principal Component Analysis (PCA), as illustrated in Figure 3, which compared the synthetic data to real data of the minor class. This comparison, spanning from −2 to 4 on both axes, demonstrated that the synthetic data closely mirrored the real data in shape and orientation, effectively replicating their variance and structure for all the three target variables. [see Supplementary Materials Section S3 for additional analyses].

After checking the augmentation validity, we fine-tuned LR, the best-performing ML model. The fine-tuning process encompassed three aspects: augmentation tuning specific to Modified SMOTE, tuning the model hyperparameters, and tuning related to feature selection. Table 3 specifies the parameters that were taken into consideration during this tuning process.

**Figure 4:**
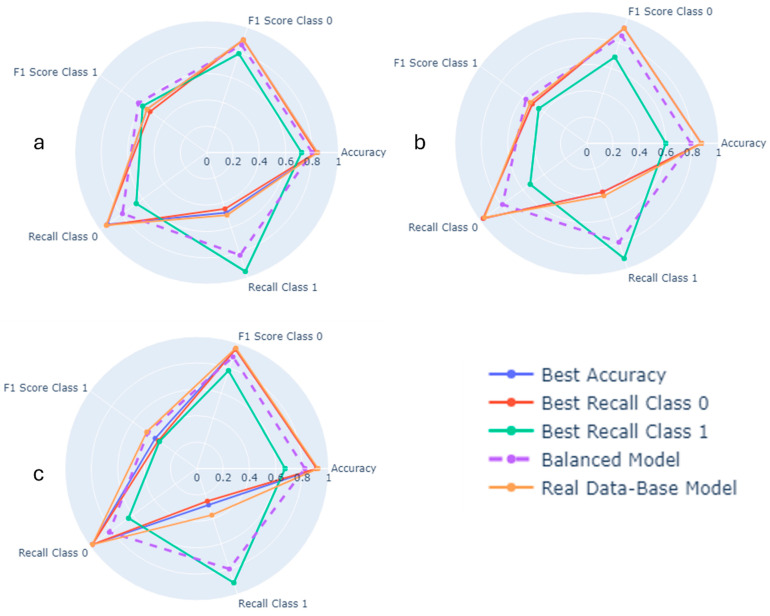
Comparison of different LR models based on different parameters: (**a**) suicide ideation; (**b**) suicide planning; and (**c**) suicide attempt.

Following the parameter tuning process, a total of 50 distinct models were developed for each target variable. The selection of the optimal model for the purposes of this study was based on a comprehensive evaluation of its performance, focusing not only on high accuracy but also on strong recall and F1 score values, particularly for class 1, which is crucial in the context of suicide prediction. To facilitate the selection of the most suitable model, Figure 4 in the study displays a radar chart that compares five different models. These models include the one with the highest accuracy, the model with the highest recall for class 1, the model with the highest recall for class 0, the base model that was trained using real data, and the balanced model, which is identified as the best in this context. The term ‘balanced’ in this case refers to the model that achieves satisfactory scores across all evaluation metrics, indicating its versatility and reliability. [See Supplementary Materials Section S4 for more information about the models].

Based on the analysis of Figure 4a, it is observed that the base model of LR using real data achieved an accuracy of 0.84. However, its recall for class 1, which is critical for predicting suicide ideation, was only 0.50. This highlights a significant limitation of the model’s predictive capability for the most important outcome. Similarly, the models with the best accuracy and the best recall for class 0 also underperformed in terms of recall for class 1. Interestingly, the model that achieved the highest recall for class 1 reached a remarkable recall of 0.95. However, this model fell short in overall accuracy (0.72) and did not perform well in recall and F1 score for the other class. In contrast, the balanced model demonstrated a more holistic performance, with a high recall for class 1 (0.82) and a satisfactory accuracy of 0.80, indicating a better equilibrium in predicting both classes. In Figure 4b, the base model using only real data showed promising accuracy (0.87) but had a low recall score of 0.42 for predicting suicide planning. Similarly, the model with the best recall score for class 0 had the same accuracy issue (0.82), but an even lower recall for class 1. In this scenario, the balanced model stood out again with a good balance of accuracy (0.79) and high recall for class 1, along with achieving the highest F1 score for class 1. The trend continues in Figure 4c, where the balanced model is the only one demonstrating satisfactory results across all metrics. This consistent performance across different targets underscores the effectiveness of the balanced model. Following this comparative analysis, the next step involves evaluating the balanced models using the ROC curve to assess their generalizability.

**Figure 5:**
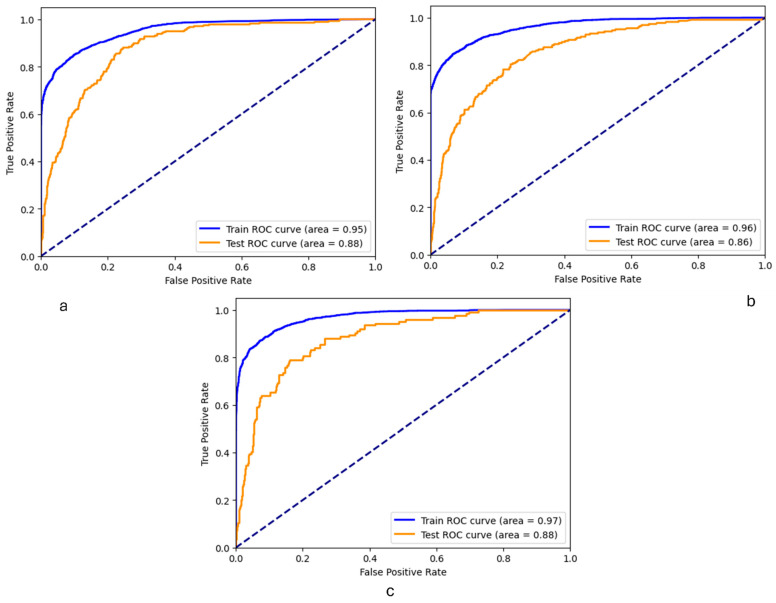
ROC curves of the training and testing of the LR models for (**a**) suicide ideation; (**b**) suicide planning; and (**c**) suicide attempt.

Figure 5 presents the ROC curves for the LR models (balanced models), specifically developed for predicting suicide ideation, planning, and attempts. The ROC curves reveal a high degree of generalization capability in these models, as indicated by the impressive Area Under the Curve (AUC) scores for both the training and testing datasets across all the three targets, implying the absence of overfitting. Achieving this balance between training performance and test data is essential for the practical application of these models in real-world settings, ensuring that the models can be trusted to predict outcomes reliably on new and unobserved datasets. Given these findings, the models were subsequently applied in a SHAP analysis to understand the significant features influencing the predictions.

**Figure 6:**
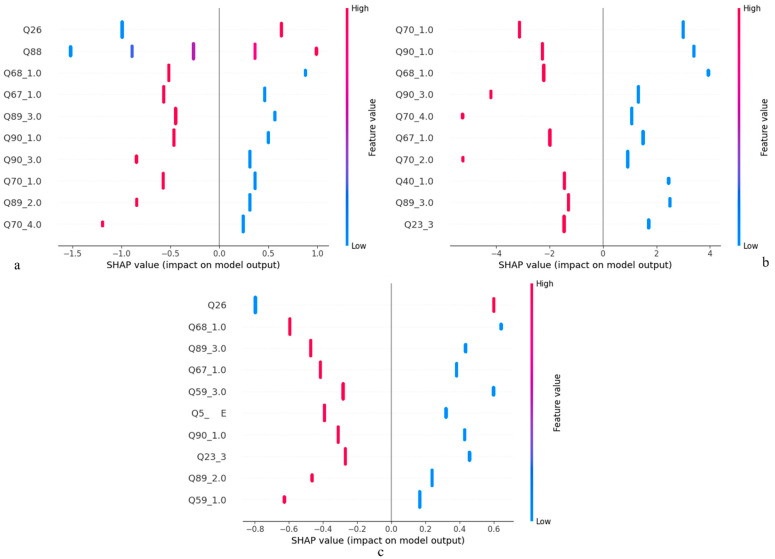
SHAP analysis of the LR models for (**a**) suicide ideation; (**b**) suicide planning; and (**c**) suicide attempt.

Figure 6 shows a SHAP summary plot that displays the impact of the top 10 features on the model output. In this Figure, the *X*–axis (SHAP value) represents the impact of a feature on the model output. A feature with a SHAP value of zero would indicate no impact on the model’s output, whereas a feature with a higher absolute value (positive or negative) has a greater impact. The *Y*–axis (Features) lists the features used in the model, and the color represents the value of the features, where one side of the color spectrum (blue) shows a low feature value, and the other side (red) shows a high feature value. In Figure 6a, features such as Q26 (indicative of sad feelings and hopelessness) and Q88 (representing mental health status during COVID-19) demonstrate that high values are associated with an increased likelihood of suicide ideation, as reflected by their positive SHAP values. Conversely, Q70_4.0, a feature indicating individuals who are not preoccupied with weight control or do not have a weight loss plan, shows negative SHAP values. This suggests that not being focused on weight control is associated with a decreased likelihood of suicide ideation. Figure 6b displays a broader range of SHAP values compared to (a), indicating that the features have a more substantial impact on the model’s output for suicide planning. High values in features such as Q90_1.0 (denoting unemployment before COVID-19) and Q70_1.0 (representing individuals actively trying to lose weight) significantly increase the likelihood of suicide planning. On the other hand, a feature like Q40_1.0, which indicates non-use of tobacco in the last 12 months, has low values that significantly reduce the likelihood of suicide planning. In Figure 6c, the influence of different features on the likelihood of a suicide attempt varies. Notably, Q26 remains a consistent and significant feature across all models, highlighting its importance in the context of suicide behaviors. Also, Q67, which pertains to sexual behavior, is identified as having a substantial impact. Additional features like Q59 (relating to being offered or sold drugs in school) and Q5 (indicating race) also emerge as important factors in a suicide attempt. The presence of Q26 across all models underlines the strong link between feelings of sadness and hopelessness, and the risk of suicide ideation, planning, and attempts. The inclusion of factors like mental health during COVID-19, weight management plans, employment status, tobacco use, drug exposure, and racial background illustrates the multifaceted nature of suicide risk factors.

## 4. Discussion

Our study shows the relationship between mental health issues, socio-economic conditions, lifestyle choices, and environmental influences, and how they contribute uniquely to the risk profile for suicidal behaviors. This nuanced understanding necessitates a multifaceted approach to suicide prevention, integrating mental health care, socio-economic support, lifestyle modification, and social policy interventions. Furthermore, the use of modified SMOTE, adds to the novelty of our study. By generating synthetic data, we addressed the challenge of imbalanced datasets. Our modified data augmentation technique significantly enhanced the performance of the ML models. The optimal ML model demonstrated high discrimination capabilities across all target variables, effectively predicting different phases of suicide risk. Table 4 presents a comparative analysis of our study’s results alongside findings from other relevant literature, providing a context for the effectiveness and advancements our approach offers in the field of suicide prediction research.

Based on Table 4, while various studies have focused on predicting suicide ideation and attempts, less attention has been given to suicide planning. In contrast, our study provides detailed predictions for suicide ideation, planning, and attempts, utilizing four augmentation techniques and five ML models, with the best model fine-tuned for optimal performance. Specifically, our models demonstrated superior recall compared to the findings in [25,41,42,43,44,45], higher accuracy than studies [29,40], and better AUC scores compared to [25,29,42]. This comprehensive approach and the resultant performance underscore the efficacy and advancement of our study in the field of suicide prediction.

Our findings have several implications. For instance, the prominence of Q26 in all models, highlighting the impact of sad feelings and hopelessness, underscores the critical need for accessible, effective mental health services. This could involve the expansion of community-based mental health programs that offer early identification and treatment for depression and other mood disorders. For instance, integrating mental health screenings into routine healthcare visits could facilitate early detection of at-risk individuals, enabling timely intervention. Additionally, the role of Q88, reflecting mental health status during COVID-19, underscores the necessity for mental health interventions to be adaptable, ensuring continuity and accessibility of support during crisis situations. Providing teletherapy options and online support groups can help maintain mental health care access during lockdowns or periods of social distancing.

The analysis also highlights socio-economic and lifestyle factors, such as unemployment (Q90_1.0) and active efforts to lose weight (Q70_1.0), as significant contributors to suicide planning. This suggests a need for comprehensive support systems that address the broader socio-economic challenges individuals face. Initiatives like job training programs and employment support services could mitigate the impact of unemployment on mental health. Similarly, public health campaigns promoting healthy eating and exercise, along with psychological support for individuals struggling with body image issues, can counteract unhealthy weight control practices. Conversely, the protective association of not focusing on weight control (Q70_4.0) and non-use of tobacco (Q40_1.0) with reduced suicide ideation and planning indicates the beneficial impact of healthy lifestyle behaviors. Encouraging these behaviors through public health initiatives, such as anti-smoking campaigns and programs that foster positive body image, could serve as preventive measures against suicide.

The influence of social and environmental factors, including drug exposure in schools (Q59) and racial background (Q5), on the likelihood of a suicide attempt, points to the need for targeted interventions. Efforts to create safe, supportive school environments, such as anti-bullying programs and drug prevention education, can reduce exposure to these risk factors. Furthermore, addressing systemic issues that contribute to racial disparities in suicide risk requires a commitment to social justice and policy reform. Implementing policies that reduce social inequities and promote inclusivity can help mitigate the impact of these factors on suicide risk.

Our study has some limitations to consider. Firstly, the representativeness of the sample may be limited, as the data only includes adolescents who attend school, potentially excluding those who do not attend or have dropped out. This exclusion could result in an underrepresentation of vulnerable groups who may have different risk profiles for suicidal behavior. Secondly, although the sample size is large, it may not be nationally representative, and the findings may not fully generalize to all adolescents across the country. Despite these limitations, our model demonstrated a promising performance, with a recall of 0.82, an accuracy of 0.80, and an AUC of 0.88, which is higher than many existing tools. However, it is important to note that this model cannot replace clinical assessment. These limitations suggest that, while our model holds promise for aiding health policymakers in designing public health interventions, further research is needed to ensure its applicability to a more comprehensive and diverse adolescent population.

## 5. Conclusions

This study presents a comprehensive and balanced ML model that excels in all key metrics for predicting suicide ideation, planning, and attempts. By utilizing a combination of data augmentation techniques, including the Modified SMOTE, and fine-tuning the best-performing model, we achieved balanced and robust outcomes. Notably, our model’s effectiveness extends to unseen data, demonstrating its reliability and applicability to real-world actions addressing mental health, socio-economic status, lifestyle, and environmental factors. By tailoring interventions to the multifaceted nature of suicide risk, leveraging community resources, and advocating for policy changes that address underlying social determinants, we encourage the development of more effective strategies to reduce suicidal behaviors and save lives.

Our model holds potential for health policymakers and professionals in designing effective public health interventions. By identifying more vulnerable sub-groups within regions, such a model allows for the implementation of tailored interventions that facilitate early identification and referral to medical treatment. It can help in the adaptation of public health strategies to meet the specific needs of individuals exhibiting high-risk suicidal behavior, ultimately improving outcomes and providing targeted support to those most in need.

## 6. Summary Points

- Sad feelings, hopelessness, sexual behavior, and being overweight were noted as some of the most important predictors;
- The optimized logistic regression model demonstrated high discrimination capabilities across all target variables;
- Encouraging these behaviors through public health initiatives, such as anti-smoking campaigns and programs that foster anti-bullying behaviors, could serve as preventive measures against suicide.

## Figures and Tables

**Table 1:** Participant characteristics.

Attribute	Overall Data	Suicide Ideation N (%)	Suicide Planning N (%)	Suicide Attempt N (%)
**Age (years)**				
12 to 17	6622 (86)	1350 (18)	1068 (14)	1348 (18)
18 and above	1070 (14)	186 (2)	138 (2)	243 (3)
**Sex**				
Male	3678 (48)	490 (6)	364 (5)	702 (9)
Female	3999 (52)	1035 (13)	832 (11)	880 (11)
**Ethnicity**				
Hispanic or Latino	2038 (26)	401 (5)	338 (4)	450 (6)
Not Hispanic	5634 (73)	1136 (15)	868 (11)	1138 (15)
**Race**				
American Indian or Alaska Native	276 (4)	50 (<1)	40 (<1)	72 (<1)
Asian	381 (5)	69 (<1)	62 (<1)	71 (<1)
African American	1301 (19)	216 (3)	177 (3)	354 (5)
Native Hawaiian or other Pacific Islander	98 (1)	14 (<1)	12 (<1)	32 (<1)
Caucasian	4335 (62)	930 (13)	702 (10)	790 (11)
Multiracial	639 (9)	165 (2)	135 (2)	130 (2)

**Table 2:** Comparing different models before data augmentation.

Models	Suicide Ideation			Suicide Planning			Suicide Attempt		
	Accuracy	Recall	Recall	Accuracy	Recall	Recall	Accuracy	Recall	Recall
		Class 0	Class 1		Class 0	Class 1		Class 0	Class 1
Random Forest	0.85	0.97	0.42	0.87	0.99	0.29	0.92	0.99	0.23
Decision Tree	0.78	0.88	0.41	0.82	0.91	0.43	0.87	0.93	0.36
Logistic Regression	0.84	0.94	0.50	0.87	0.96	0.42	0.92	0.98	0.37
Support Vector Machine	0.84	0.96	0.41	0.86	0.98	0.30	0.91	0.99	0.20
Extreme Gradient Boosting	0.84	0.94	0.48	0.87	0.96	0.45	0.92	0.98	0.35

**Table 3:** Parameters considered in the fine-tuning process of LR model with Modified SMOTE.

	Parameters	Different Values
Modified SMOTE	Sample strategy	0.5, 0.6, 0.7, 0.75, 0.9, 1
Model—Logistic Regression	C	0.01, 0.1, 0.5, 1, 10, 100
	Solver	‘lbfgs’, ‘liblinear’
	Class weights	(0: 1, 1: 1), (0: 1, 1: 2), (0: 1, 1: 2.5), (0: 1, 1: 3), (0: 1, 1: 3.5), (0: 1, 1: 5), (0: 1, 1: 10)
Feature Selection	Significant features	Recursive Feature Elimination

**Table 4:** Comparing our model with related literature in suicide prediction field.

Ref.	Study Data	SI	SP	SA	Best Model	Results	Important Factors
**Our model**	Adolescent Behaviors and Experiences Survey (ABES).	**x**	**x**	**x**	LR	Recall: 0.82 Accuracy: 0.80 AUC: 0.88	Sad feelings, hopelessness, experiences during COVID-19, sexual behavior, body weight
[29]	U.S. veterans’ recordings	x			RF	Recall: 0.84 Accuracy: 0.72 AUC: 0.80	Delta energy entropy, Delta energy, Energy contour
[40]	Social media (Reddit) contents	x			Navie bayes	Recall: 0.87 Accuracy: 0.74 AUC: NR	50 linguistic features
[41]	Human-annotated dataset	x			LR	Recall: 0.79 Accuracy: 0.79 AUC: NR	NR
[25]	Generalized q-sampling imaging (GQI) dataset	x			XGB	Recall: 0.73 Accuracy: 0.68 AUC: 0.84	NR
[42]	Psychotherapy dyads	x			XGB	Recall: 0.66 Accuracy: NR AUC: 0.82	NR
[43]	Nationwide survey data (Norwegian adolescents)			x	XGB	Recall: 0.77 Accuracy: NR AUC: 0.92	Sadness and depression, contacting a psychologist, feeling worthless
[44]	MGB Research Patient Data Registry (RPDR)			x	regularized Cox	Recall: 0.70 Accuracy: 0.93 AUC: NR	Suicide ideation, mood disorder, age
[45]	Korea Youth Risk Behavior Survey (KYRBS)			x	XGB	Recall: 0.61 Accuracy: 0.97 AUC: NR	Suicide ideation, suicide planning, grade

SI: suicide ideation; SP: suicide planning; SA: suicide attempt; NR: not reported; x: reported.

## Data Availability

The data used in this study is available from https://www.cdc.gov/healthyyouth/data/abes/data.htm.

## Supplementary Materials

The following supporting information can be downloaded at: https://www.mdpi.com/article/10.3390/healthcare12131262/s1, Table S1: The methods employed to deal with missing values. Table S2: The results of the fine tuning process to develop the ML models for suicide ideation prediction. Table S3: The results of the fine tuning process to develop the ML models for suicide planning prediction. Table S4: The results of the fine tuning process to develop the ML models for suicide attempt prediction. Figure S1: Box plot of height and weight of the participents. Figure S2: Counts of target varables grouped by sex. Figure S3: Top 10 binary features in terms of proportion. Figure S4: Visualization of the dataset’s imbalance utilizing t-SNE. Figure S5: Visualization of the dataset’s imbalance utilizing PCA. Figure S6: Parallel coordinate plot for the targets: Suicide ideation to attempt through planning. Figure S7: The overall f2 score of minor class for different ML models after applying different augmentation techniques on target features: (a) suicide ideation, (b) suicide planning, and (c) suicide attempt. Figure S8: The overall precision score of minor class for different ML models after applying different augmentation techniques on target features: (a) suicide ideation, (b) suicide planning, and (c) suicide attempt. Figure S9: Comprehensive comparison between mean of the real and synthetic data (log): (a) suicide ideation, (b) suicide planning, and (c) suicide attempt. Figure S10: Comprehensive comparison between std of the real and synthetic data (log): (a) suicide ideation, (b) suicide planning, and (c) suicide attempt. Figure S11: Comprehensive comparison between the real and fake data considering t-sne: (a) suicide ideation, (b) suicide planning, and (c) suicide attempt. Figure S12: Comprehensive comparison between the distributions of real and fake data across age, sex, and grade: (a) suicide ideation, (b) suicide planning, and (c) suicide attempt. Figure S13: Scatter plots representing the trade-off between accuracy and recall for Class 1 across different models for (a) suicide ideation, (b) suicide planning, and (c) suicide attempt. Figure S14: Violin plots representing the distribution of model accuracy across different class weights for (a) suicide ideation, (b) suicide planning, and (c) suicide attempt. Figure S15: Violin plots representing the distribution of recall class 1 across different class weights for (a) suicide ideation, (b) suicide planning, and (c) suicide attempt.
