# Supplementary material for: Predicting Suicidal Ideation, Planning, and Attempts among the Adolescent Population of the United States
Source: Healthcare (Basel). 2024 Jun 25;12(13):1262. doi: 10.3390/healthcare12131262 (PMC11241284; doi:10.3390/healthcare12131262)
Supplement: Supplementary file 1 [file healthcare-12-01262-s001.zip › healthcare-3034662-supplementary.pdf]

Supplement S1

Table S1: The methods employed to deal with missing values

Features	Methods
Q1, Q3	Median (After checking the distribution, which was skewed)
Q2, Q4, Q8, Q9, Q24, Q25, Q26, Q70 to Q99	Mode
Q6, Q7, Q69, Q35	Group-wise imputation
Q12-22, Q23, Q31-34. Q41-45, Q46-49, Q50-58	Multivariate Imputation by Chained Equations (MICE)
Q5, Q35, Q36, Q37	Random imputation based on distribution
Q10, Q11, Q60-68	Clustering
Q38, Q39, Q40, Q59	Presictive models (Random Forest and Logistic Regression)

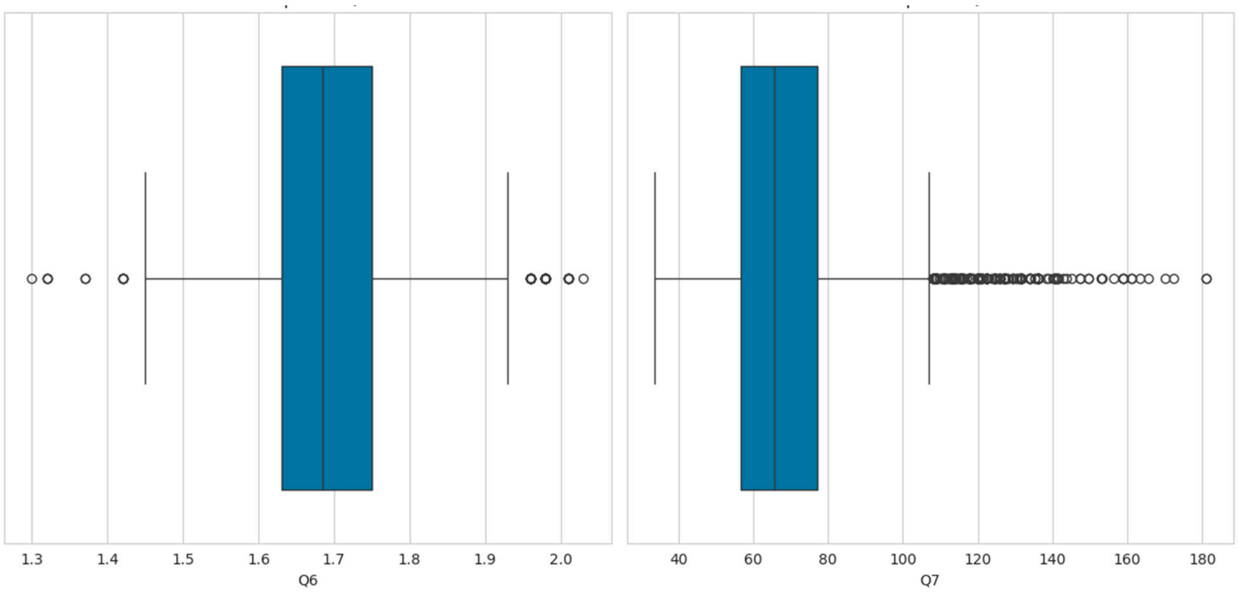

Figure S1: Box plot of height and weight of the participents

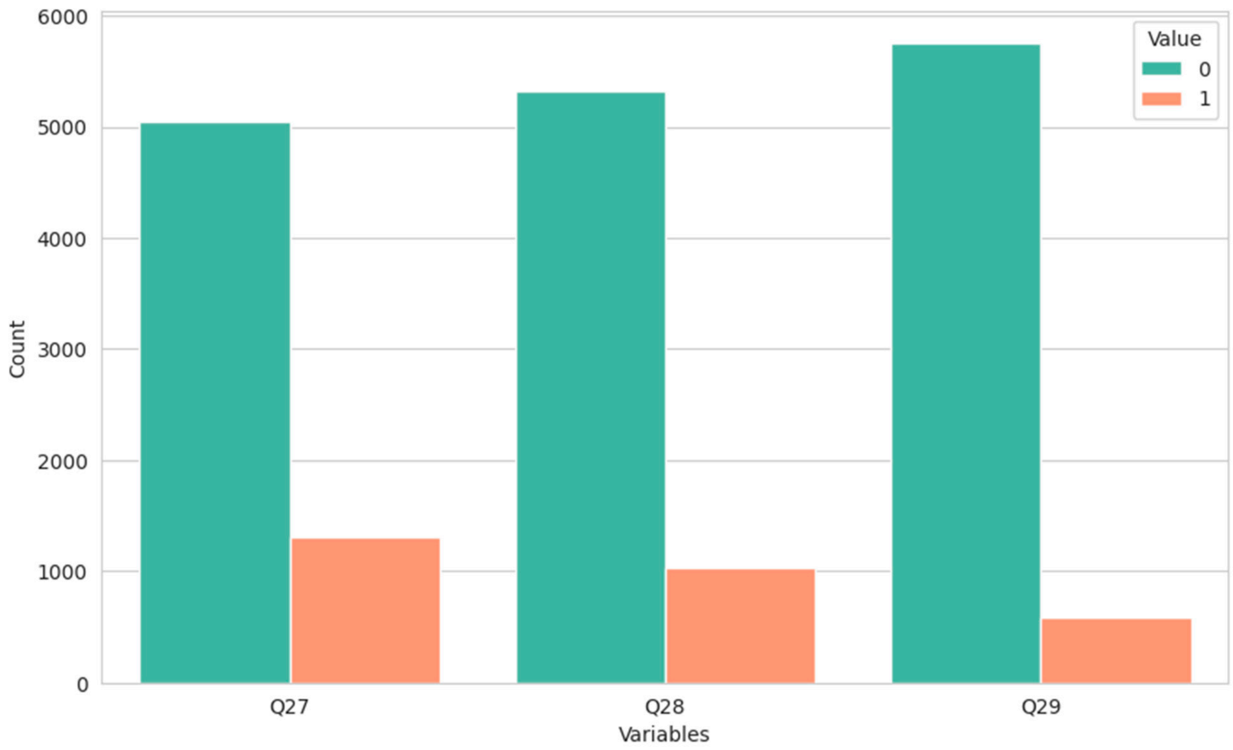

Figure S2: Counts of target varables grouped by sex

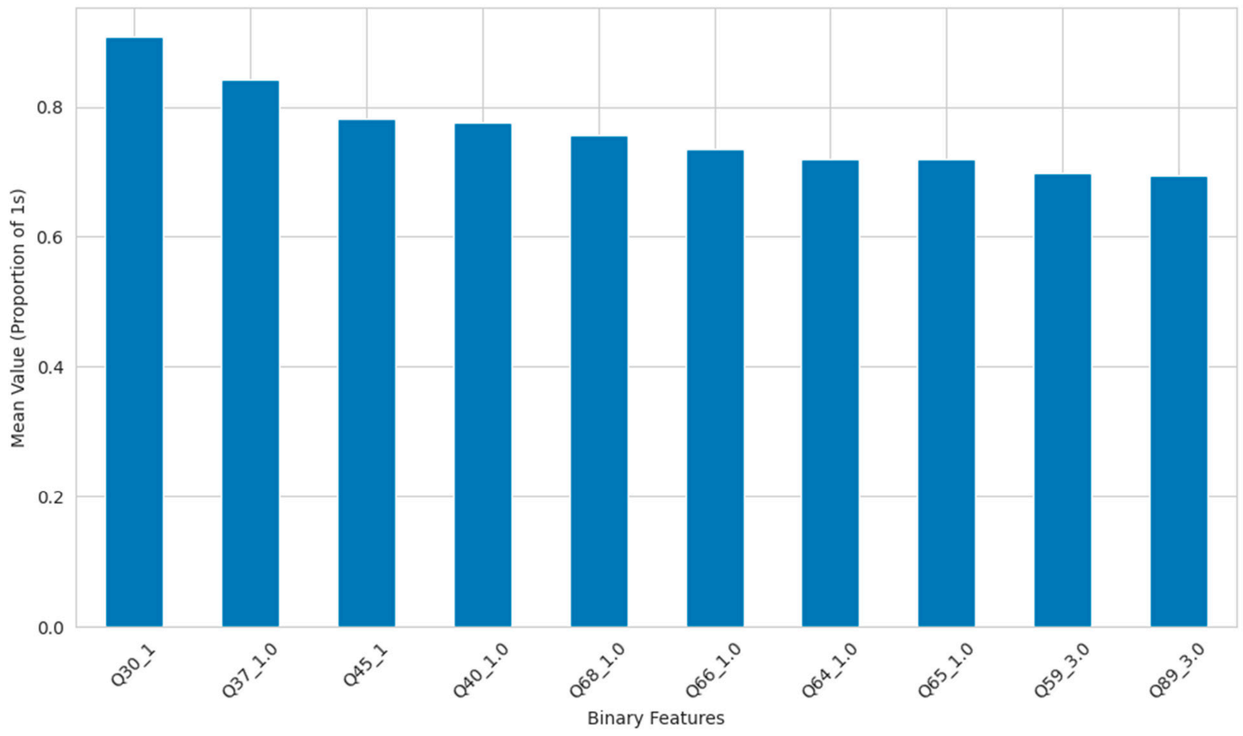

Figure S3: Top 10 binary features in terms of proportion

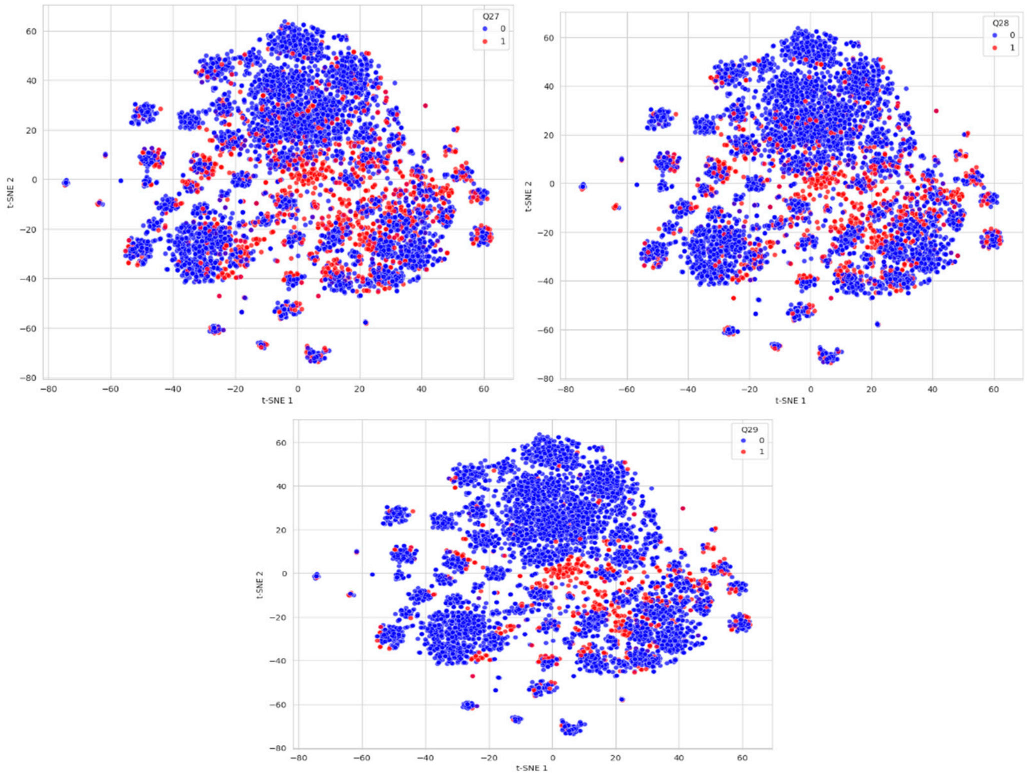

Figure S4: Visualization of the dataset's imbalance utilizing t-SNE

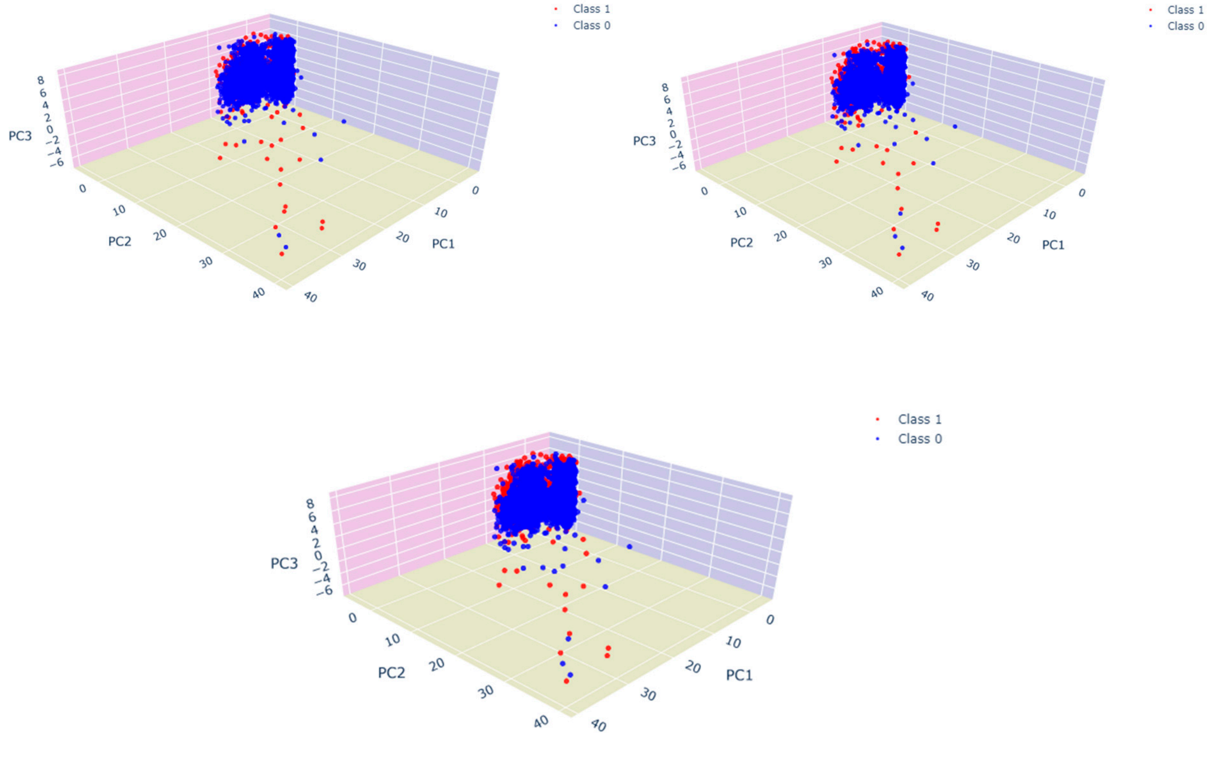

Figure S5: Visualization of the dataset's imbalance utilizing PCA

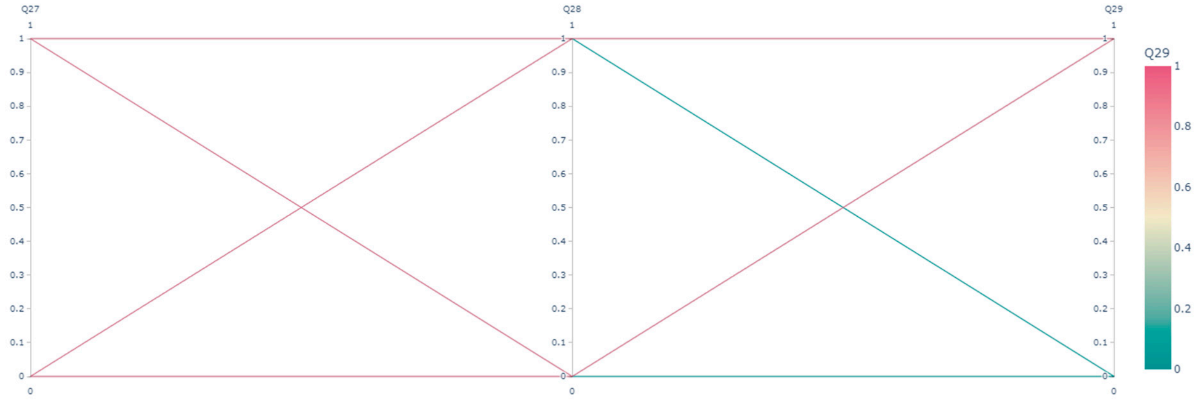

Figure S6: Parallel coordinate plot for the targets: Suicide ideation to attempt through planning

Supplement S2

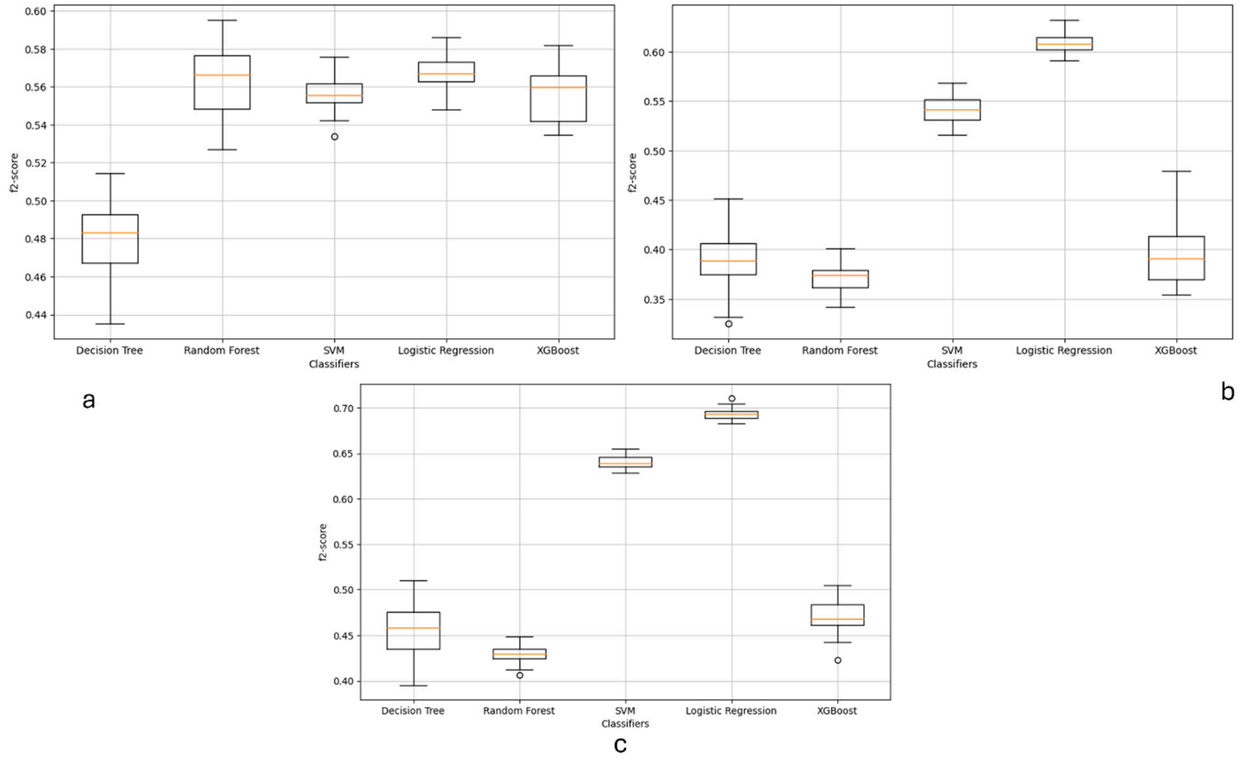

Figure S7: The overall f2 score of minor class for different ML models after applying different augmentation techniques on target features: a) suicide ideation, b) suicide planning, and c) suicide attempt

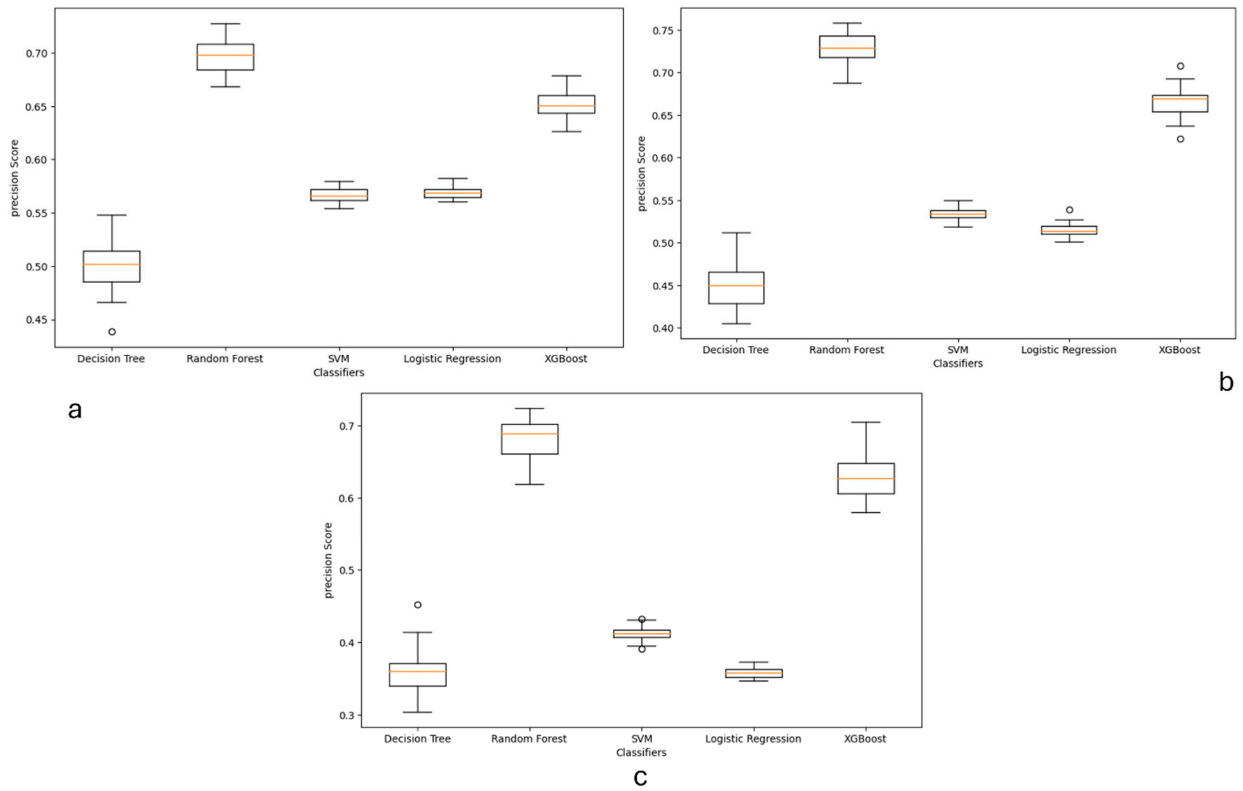

Figure S8: The overall precision score of minor class for different ML models after applying different augmentation techniques on target features: a) suicide ideation, b) suicide planning, and c) suicide attempt

Supplement S3

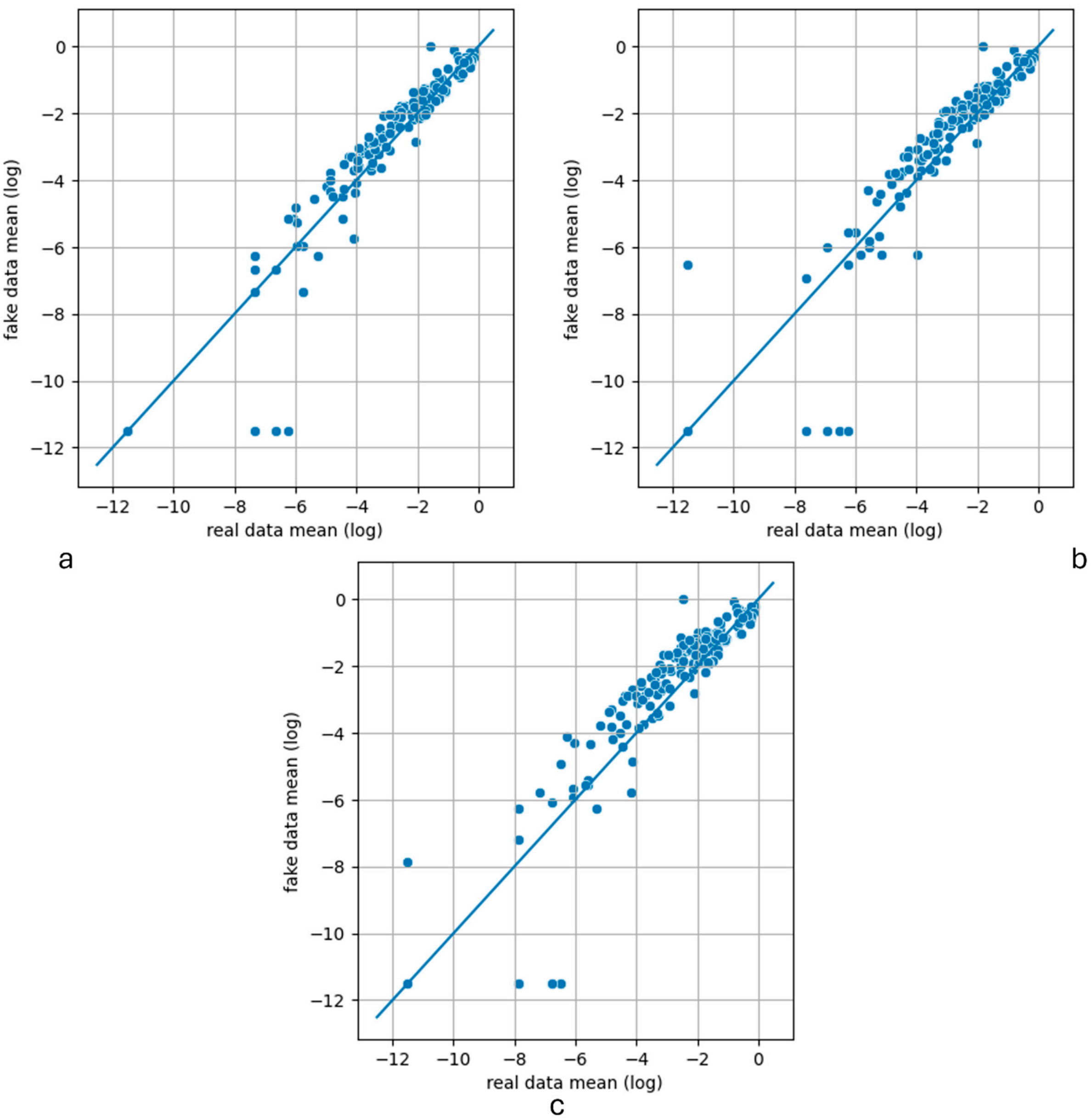

Figure S9: Comprehensive comparison between mean of the real and synthetic data (log): a) suicide ideation, b) suicide planning, and c) suicide attempt

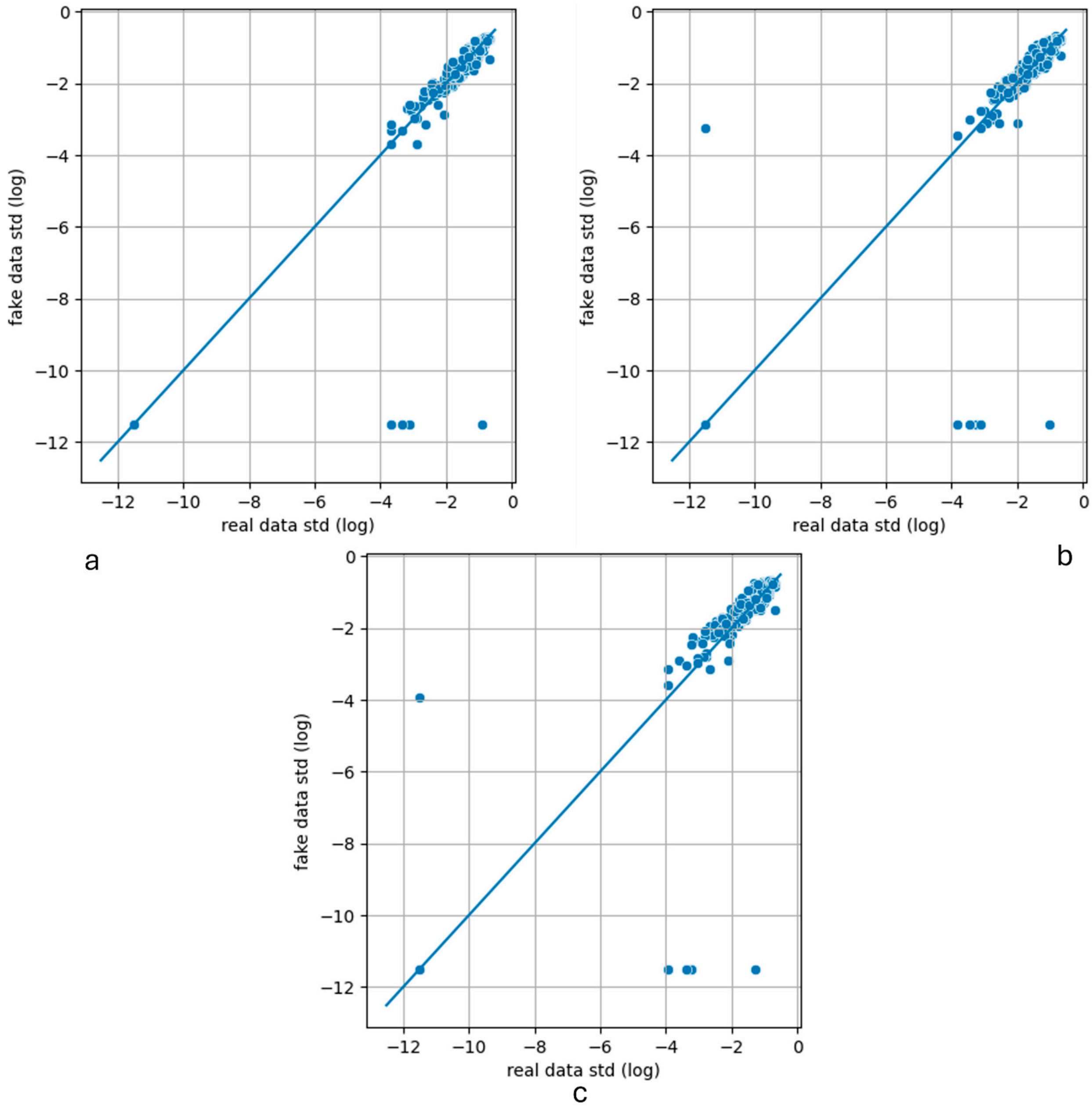

Figure S10: Comprehensive comparison between std of the real and synthetic data (log): a) suicide ideation, b) suicide planning, and c) suicide attempt

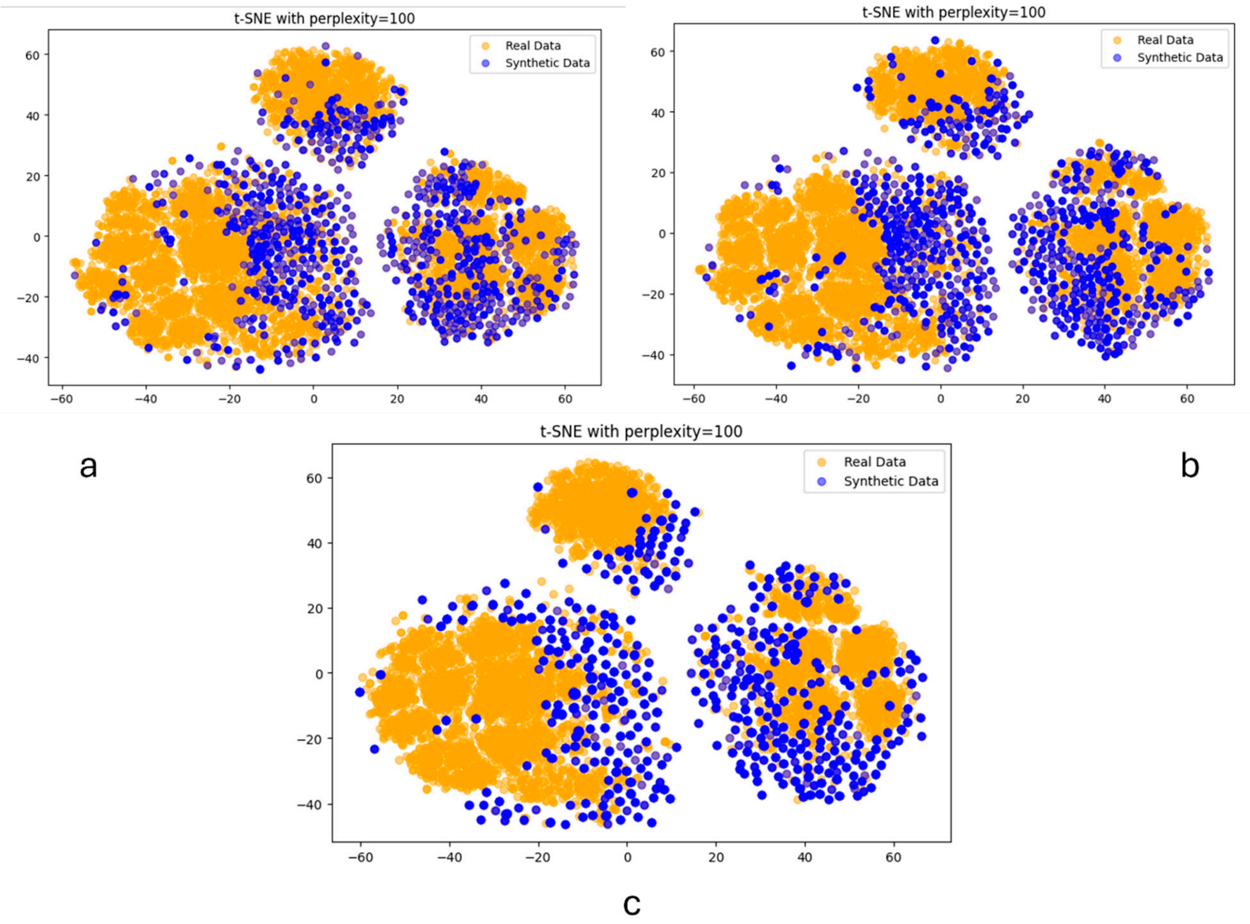

Figure S11: Comprehensive comparison between the real and fake data considering t-sne: a) suicide ideation, b) suicide planning, and c) suicide attempt

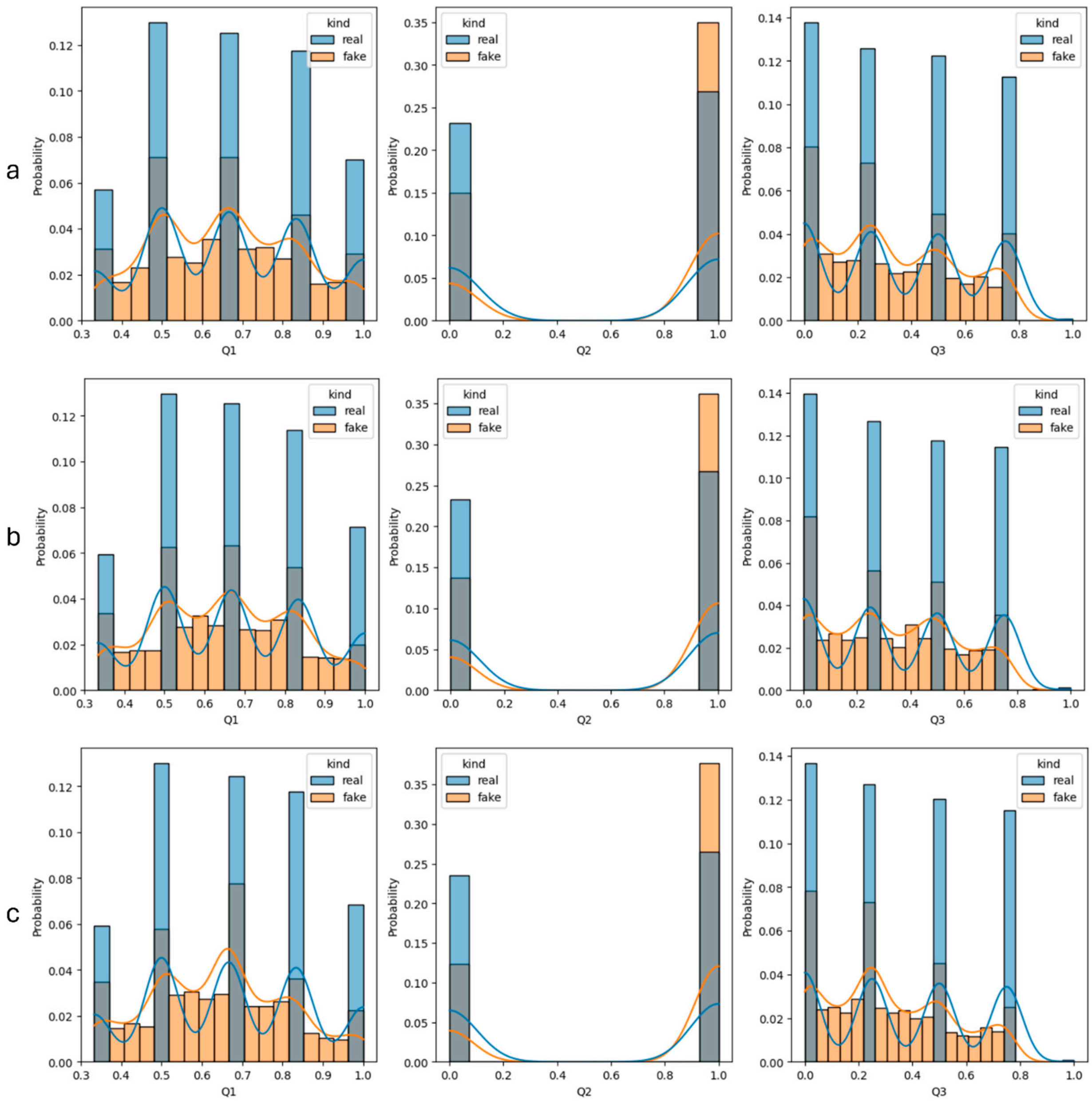

Figure S12: Comprehensive comparison between the distributions of real and fake data across age, sex, and grade: a) suicide ideation, b) suicide planning, and c) suicide attempt

Supplement S4

Table S1: The results of the fine tuning process to develop the ML models for suicide ideation prediction

Sampling Strategy	Class Weights	Best Parameters	Accuracy	Recall Class 0	Recall Class 1	Precision Class 0	Precision Class 1	F1 Score Class 0	F1 Score Class 1	F2 Score	AUC
0.50	{0: 1, 1: 1}	{'C': 100, 'solver': 'liblinear'}	0.83	0.94	0.45	0.86	0.67	0.90	0.54	0.48	0.87

0.50	{0: 1, 1: 2}	{'C': 100, 'solver': 'lbfgs'}	0.83	0.87	0.69	0.91	0.59	0.89	0.64	0.67	0.87
0.50	{0: 1, 1: 2.5}	{'C': 1, 'solver': 'lbfgs'}	0.82	0.84	0.74	0.92	0.57	0.88	0.64	0.70	0.89
0.50	{0: 1, 1: 3}	{'C': 100, 'solver': 'liblinear'}	0.81	0.81	0.79	0.93	0.54	0.87	0.64	0.72	0.87
0.50	{0: 1, 1: 3.5}	{'C': 100, 'solver': 'lbfgs'}	0.80	0.79	0.83	0.94	0.52	0.86	0.64	0.74	0.89
0.50	{0: 1, 1: 5}	{'C': 100, 'solver': 'liblinear'}	0.77	0.73	0.88	0.96	0.48	0.83	0.62	0.75	0.88
0.50	{0: 1, 1: 10}	{'C': 100, 'solver': 'liblinear'}	0.72	0.66	0.93	0.97	0.43	0.78	0.59	0.76	0.87
0.60	{0: 1, 1: 1}	{'C': 100, 'solver': 'liblinear'}	0.83	0.94	0.44	0.86	0.67	0.90	0.53	0.47	0.87
0.60	{0: 1, 1: 2}	{'C': 100, 'solver': 'liblinear'}	0.83	0.87	0.69	0.91	0.59	0.89	0.64	0.67	0.87
0.60	{0: 1, 1: 2.5}	{'C': 10, 'solver': 'lbfgs'}	0.82	0.84	0.74	0.92	0.56	0.88	0.64	0.70	0.88
0.60	{0: 1, 1: 3}	{'C': 100, 'solver': 'liblinear'}	0.80	0.81	0.79	0.93	0.53	0.86	0.64	0.72	0.88
0.60	{0: 1, 1: 3.5}	{'C': 10, 'solver': 'lbfgs'}	0.78	0.76	0.83	0.94	0.49	0.84	0.62	0.73	0.87
0.60	{0: 1, 1: 5}	{'C': 10, 'solver': 'lbfgs'}	0.77	0.74	0.88	0.96	0.49	0.84	0.63	0.76	0.89
0.60	{0: 1, 1: 10}	{'C': 10, 'solver': 'lbfgs'}	0.72	0.66	0.95	0.98	0.44	0.79	0.60	0.77	0.89
0.64	{0: 1, 1: 1}	{'C': 0.5, 'solver': 'liblinear'}	0.84	0.93	0.48	0.87	0.67	0.90	0.56	0.51	0.88
0.64	{0: 1, 1: 2}	{'C': 100, 'solver': 'liblinear'}	0.84	0.87	0.70	0.91	0.61	0.89	0.65	0.68	0.88
0.64	{0: 1, 1: 2.5}	{'C': 100, 'solver': 'lbfgs'}	0.82	0.83	0.75	0.92	0.56	0.88	0.64	0.70	0.87
0.64	{0: 1, 1: 3}	{'C': 100, 'solver': 'lbfgs'}	0.80	0.80	0.79	0.93	0.52	0.86	0.63	0.71	0.87
0.64	{0: 1, 1: 3.5}	{'C': 100, 'solver': 'lbfgs'}	0.77	0.76	0.82	0.94	0.49	0.84	0.61	0.72	0.87
0.64	{0: 1, 1: 5}	{'C': 100, 'solver': 'lbfgs'}	0.77	0.74	0.88	0.96	0.48	0.83	0.62	0.76	0.88
0.64	{0: 1, 1: 10}	{'C': 100, 'solver': 'liblinear'}	0.73	0.67	0.94	0.98	0.44	0.79	0.60	0.77	0.89
0.70	{0: 1, 1: 1}	{'C': 1, 'solver': 'liblinear'}	0.83	0.93	0.47	0.86	0.66	0.90	0.55	0.50	0.88
0.70	{0: 1, 1: 2}	{'C': 100, 'solver': 'liblinear'}	0.83	0.86	0.70	0.91	0.59	0.89	0.64	0.67	0.87
0.70	{0: 1, 1: 2.5}	{'C': 100, 'solver': 'liblinear'}	0.82	0.84	0.75	0.92	0.56	0.88	0.64	0.70	0.87

0.70	{0: 1, 1: 3}	{'C': 10, 'solver': 'liblinear'}	0.80	0.80	0.79	0.93	0.52	0.86	0.63	0.71	0.87
0.70	{0: 1, 1: 3.5}	{'C': 100, 'solver': 'lbfgs'}	0.78	0.76	0.83	0.94	0.49	0.84	0.62	0.73	0.87
0.70	{0: 1, 1: 5}	{'C': 1, 'solver': 'liblinear'}	0.75	0.70	0.91	0.96	0.46	0.81	0.61	0.76	0.88
0.70	{0: 1, 1: 10}	{'C': 10, 'solver': 'liblinear'}	0.73	0.67	0.93	0.97	0.44	0.79	0.60	0.76	0.88
0.75	{0: 1, 1: 1}	{'C': 100, 'solver': 'lbfgs'}	0.83	0.94	0.45	0.86	0.67	0.90	0.53	0.48	0.87
0.75	{0: 1, 1: 2}	{'C': 100, 'solver': 'lbfgs'}	0.83	0.87	0.69	0.91	0.59	0.89	0.64	0.67	0.87
0.75	{0: 1, 1: 2.5}	{'C': 100, 'solver': 'liblinear'}	0.82	0.84	0.75	0.92	0.56	0.88	0.64	0.70	0.87
0.75	{0: 1, 1: 3}	{'C': 100, 'solver': 'liblinear'}	0.80	0.80	0.80	0.93	0.53	0.86	0.64	0.72	0.87
0.75	{0: 1, 1: 3.5}	{'C': 10, 'solver': 'lbfgs'}	0.78	0.76	0.83	0.94	0.49	0.84	0.62	0.73	0.87
0.75	{0: 1, 1: 5}	{'C': 10, 'solver': 'lbfgs'}	0.75	0.71	0.89	0.96	0.46	0.81	0.61	0.75	0.87
0.75	{0: 1, 1: 10}	{'C': 10, 'solver': 'liblinear'}	0.73	0.67	0.93	0.97	0.44	0.79	0.60	0.76	0.89
0.90	{0: 1, 1: 1}	{'C': 0.01, 'solver': 'liblinear'}	0.83	0.89	0.63	0.90	0.61	0.89	0.62	0.63	0.88
0.90	{0: 1, 1: 2}	{'C': 0.01, 'solver': 'lbfgs'}	0.81	0.81	0.77	0.93	0.54	0.87	0.63	0.71	0.87
0.90	{0: 1, 1: 2.5}	{'C': 0.1, 'solver': 'liblinear'}	0.80	0.79	0.82	0.94	0.52	0.86	0.64	0.73	0.88
0.90	{0: 1, 1: 3}	{'C': 0.01, 'solver': 'lbfgs'}	0.75	0.71	0.87	0.95	0.46	0.82	0.60	0.74	0.87
0.90	{0: 1, 1: 3.5}	{'C': 10, 'solver': 'liblinear'}	0.78	0.76	0.83	0.94	0.49	0.84	0.62	0.73	0.87
0.90	{0: 1, 1: 5}	{'C': 10, 'solver': 'lbfgs'}	0.77	0.73	0.89	0.96	0.48	0.83	0.63	0.76	0.88
0.90	{0: 1, 1: 10}	{'C': 100, 'solver': 'lbfgs'}	0.72	0.66	0.95	0.98	0.44	0.79	0.60	0.77	0.89
1.00	{0: 1, 1: 1}	{'C': 0.01, 'solver': 'liblinear'}	0.83	0.88	0.65	0.90	0.60	0.89	0.63	0.64	0.88
1.00	{0: 1, 1: 2}	{'C': 0.01, 'solver': 'lbfgs'}	0.79	0.78	0.81	0.94	0.51	0.85	0.62	0.72	0.88
1.00	{0: 1, 1: 2.5}	{'C': 0.01, 'solver': 'lbfgs'}	0.78	0.75	0.87	0.95	0.49	0.84	0.63	0.76	0.88
1.00	{0: 1, 1: 3}	{'C': 0.01, 'solver': 'lbfgs'}	0.75	0.71	0.89	0.96	0.46	0.81	0.60	0.75	0.87
1.00	{0: 1, 1: 3.5}	{'C': 0.01, 'solver': 'liblinear'}	0.72	0.66	0.92	0.97	0.43	0.79	0.59	0.75	0.87

1.00	{0: 1, 1: 5}	{'C': 0.01, 'solver': 'liblinear'}	0.71	0.64	0.93	0.97	0.42	0.78	0.58	0.75	0.87
1.00	{0: 1, 1: 10}	{'C': 100, 'solver': 'lbfgs'}	0.72	0.66	0.94	0.98	0.44	0.79	0.60	0.77	0.89

Table S2: The results of the fine tuning process to develop the ML models for suicide planning prediction

**Sampling Strategy**	**Class Weights**	**Best Parameters**	**Accuracy**	**Recall Class 0**	**Recall Class 1**	**Precision Class 0**	**Precision Class 1**	**F1 Score Class 0**	**F1 Score Class 1**	**F2 Score**	**AUC**
0.50	{0: 1, 1: 1}	{'C': 100, 'solver': 'liblinear'}	0.87	0.97	0.39	0.88	0.73	0.92	0.51	0.43	0.87
0.50	{0: 1, 1: 2}	{'C': 10, 'solver': 'lbfgs'}	0.85	0.91	0.55	0.90	0.57	0.91	0.56	0.56	0.86
0.50	{0: 1, 1: 2.5}	{'C': 100, 'solver': 'lbfgs'}	0.84	0.89	0.62	0.92	0.54	0.90	0.58	0.60	0.85
0.50	{0: 1, 1: 3}	{'C': 100, 'solver': 'lbfgs'}	0.83	0.86	0.66	0.92	0.51	0.89	0.58	0.62	0.85
0.50	{0: 1, 1: 3.5}	{'C': 10, 'solver': 'lbfgs'}	0.81	0.83	0.71	0.93	0.47	0.88	0.57	0.64	0.86
0.50	{0: 1, 1: 5}	{'C': 100, 'solver': 'liblinear'}	0.78	0.78	0.78	0.94	0.43	0.85	0.56	0.67	0.85
0.50	{0: 1, 1: 10}	{'C': 100, 'solver': 'liblinear'}	0.67	0.63	0.88	0.96	0.34	0.76	0.49	0.67	0.85
0.60	{0: 1, 1: 1}	{'C': 100, 'solver': 'lbfgs'}	0.86	0.96	0.36	0.87	0.67	0.92	0.47	0.39	0.85
0.60	{0: 1, 1: 2}	{'C': 100, 'solver': 'liblinear'}	0.85	0.91	0.57	0.91	0.57	0.91	0.57	0.57	0.86
0.60	{0: 1, 1: 2.5}	{'C': 100, 'solver': 'liblinear'}	0.84	0.89	0.63	0.92	0.54	0.90	0.58	0.61	0.85
0.60	{0: 1, 1: 3}	{'C': 100, 'solver': 'lbfgs'}	0.83	0.86	0.68	0.93	0.51	0.89	0.58	0.64	0.85
0.60	{0: 1, 1: 3.5}	{'C': 100, 'solver': 'lbfgs'}	0.81	0.83	0.71	0.93	0.48	0.88	0.57	0.65	0.85
0.60	{0: 1, 1: 5}	{'C': 100, 'solver': 'liblinear'}	0.79	0.79	0.79	0.95	0.44	0.86	0.57	0.69	0.87
0.60	{0: 1, 1: 10}	{'C': 10, 'solver': 'lbfgs'}	0.68	0.63	0.88	0.96	0.34	0.76	0.49	0.67	0.86
0.67	{0: 1, 1: 1}	{'C': 100, 'solver': 'lbfgs'}	0.86	0.96	0.37	0.88	0.66	0.92	0.47	0.40	0.85
0.67	{0: 1, 1: 2}	{'C': 0.01, 'solver': 'lbfgs'}	0.82	0.86	0.66	0.92	0.50	0.89	0.57	0.62	0.85
0.67	{0: 1, 1: 2.5}	{'C': 0.01, 'solver': 'lbfgs'}	0.81	0.83	0.70	0.93	0.48	0.88	0.57	0.64	0.85
0.67	{0: 1, 1: 3}	{'C': 100, 'solver': 'liblinear'}	0.83	0.86	0.68	0.93	0.50	0.89	0.58	0.64	0.85
0.67	{0: 1, 1: 3.5}	{'C': 100, 'solver': 'liblinear'}	0.81	0.84	0.70	0.93	0.48	0.88	0.57	0.64	0.85
0.67	{0: 1, 1: 5}	{'C': 100, 'solver': 'lbfgs'}	0.77	0.77	0.77	0.94	0.42	0.85	0.55	0.66	0.85
0.67	{0: 1, 1: 10}	{'C': 100, 'solver': 'liblinear'}	0.70	0.66	0.89	0.96	0.36	0.78	0.51	0.69	0.86
0.70	{0: 1, 1: 1}	{'C': 100, 'solver': 'liblinear'}	0.85	0.96	0.34	0.87	0.66	0.91	0.45	0.38	0.86
0.70	{0: 1, 1: 2}	{'C': 0.01, 'solver': 'lbfgs'}	0.82	0.86	0.67	0.92	0.50	0.89	0.57	0.63	0.85
0.70	{0: 1, 1: 2.5}	{'C': 0.01, 'solver': 'lbfgs'}	0.81	0.83	0.70	0.93	0.47	0.88	0.56	0.64	0.85
0.70	{0: 1, 1: 3}	{'C': 0.01, 'solver': 'lbfgs'}	0.79	0.80	0.74	0.93	0.44	0.86	0.55	0.65	0.85

0.70	{0: 1, 1: 3.5}	{'C': 0.01, 'solver': 'lbfgs'}	0.76	0.76	0.76	0.94	0.40	0.84	0.53	0.65	0.85
0.70	{0: 1, 1: 5}	{'C': 100, 'solver': 'liblinear'}	0.77	0.77	0.77	0.94	0.42	0.85	0.54	0.66	0.85
0.70	{0: 1, 1: 10}	{'C': 100, 'solver': 'liblinear'}	0.70	0.66	0.88	0.96	0.36	0.78	0.51	0.68	0.87
0.75	{0: 1, 1: 1}	{'C': 100, 'solver': 'liblinear'}	0.85	0.96	0.35	0.87	0.66	0.92	0.46	0.39	0.86
0.75	{0: 1, 1: 2}	{'C': 0.01, 'solver': 'lbfgs'}	0.83	0.86	0.68	0.93	0.50	0.89	0.58	0.63	0.85
0.75	{0: 1, 1: 2.5}	{'C': 0.01, 'solver': 'lbfgs'}	0.80	0.82	0.71	0.93	0.47	0.87	0.56	0.65	0.85
0.75	{0: 1, 1: 3}	{'C': 0.01, 'solver': 'lbfgs'}	0.78	0.79	0.74	0.93	0.43	0.86	0.54	0.65	0.85
0.75	{0: 1, 1: 3.5}	{'C': 0.01, 'solver': 'lbfgs'}	0.76	0.76	0.76	0.94	0.40	0.84	0.53	0.64	0.85
0.75	{0: 1, 1: 5}	{'C': 100, 'solver': 'lbfgs'}	0.77	0.77	0.78	0.94	0.42	0.85	0.55	0.67	0.85
0.75	{0: 1, 1: 10}	{'C': 100, 'solver': 'lbfgs'}	0.67	0.63	0.88	0.96	0.34	0.76	0.49	0.66	0.85
0.90	{0: 1, 1: 1}	{'C': 0.01, 'solver': 'lbfgs'}	0.86	0.94	0.47	0.89	0.62	0.92	0.54	0.50	0.85
0.90	{0: 1, 1: 2}	{'C': 0.01, 'solver': 'lbfgs'}	0.82	0.84	0.69	0.93	0.49	0.88	0.57	0.64	0.85
0.90	{0: 1, 1: 2.5}	{'C': 0.01, 'solver': 'lbfgs'}	0.79	0.81	0.71	0.93	0.45	0.87	0.55	0.64	0.85
0.90	{0: 1, 1: 3}	{'C': 0.01, 'solver': 'lbfgs'}	0.77	0.78	0.75	0.94	0.42	0.85	0.54	0.65	0.85
0.90	{0: 1, 1: 3.5}	{'C': 0.01, 'solver': 'lbfgs'}	0.76	0.75	0.79	0.94	0.40	0.84	0.53	0.66	0.85
0.90	{0: 1, 1: 5}	{'C': 0.01, 'solver': 'liblinear'}	0.67	0.62	0.90	0.97	0.34	0.75	0.49	0.67	0.84
0.90	{0: 1, 1: 10}	{'C': 0.01, 'solver': 'liblinear'}	0.62	0.55	0.92	0.97	0.30	0.70	0.46	0.65	0.84
1.00	{0: 1, 1: 1}	{'C': 0.01, 'solver': 'lbfgs'}	0.85	0.93	0.47	0.89	0.58	0.91	0.52	0.49	0.85
1.00	{0: 1, 1: 2}	{'C': 0.01, 'solver': 'lbfgs'}	0.81	0.84	0.69	0.93	0.48	0.88	0.57	0.63	0.85
1.00	{0: 1, 1: 2.5}	{'C': 0.01, 'solver': 'lbfgs'}	0.80	0.81	0.73	0.93	0.45	0.87	0.56	0.65	0.85
1.00	{0: 1, 1: 3}	{'C': 0.01, 'solver': 'lbfgs'}	0.77	0.77	0.75	0.94	0.42	0.85	0.54	0.65	0.85
1.00	{0: 1, 1: 3.5}	{'C': 0.01, 'solver': 'liblinear'}	0.69	0.65	0.88	0.96	0.35	0.78	0.50	0.67	0.84
1.00	{0: 1, 1: 5}	{'C': 0.01, 'solver': 'liblinear'}	0.66	0.61	0.90	0.97	0.33	0.75	0.48	0.67	0.84
1.00	{0: 1, 1: 10}	{'C': 0.01, 'solver': 'liblinear'}	0.60	0.53	0.92	0.97	0.30	0.69	0.45	0.65	0.84

Table S3: The results of the fine tuning process to develop the ML models for suicide attempt prediction

Sampling Strategy	Class Weights	Best Parameters	Accuracy	Recall Class 0	Recall Class 1	Precision Class 0	Precision Class 1	F1 Score Class 0	F1 Score Class 1	F2 Score	AUC
0.50	{0: 1, 1: 1}	{'C': 100, 'solver': 'liblinear'}	0.91	0.98	0.27	0.92	0.56	0.95	0.36	0.30	0.89
0.50	{0: 1, 1: 2}	{'C': 0.01, 'solver': 'lbfgs'}	0.89	0.93	0.54	0.95	0.45	0.94	0.49	0.52	0.88
0.50	{0: 1, 1: 2.5}	{'C': 0.01, 'solver': 'lbfgs'}	0.88	0.90	0.69	0.96	0.44	0.93	0.54	0.62	0.88
0.50	{0: 1, 1: 3}	{'C': 0.01, 'solver': 'lbfgs'}	0.86	0.88	0.69	0.96	0.38	0.92	0.49	0.59	0.88
0.50	{0: 1, 1: 3.5}	{'C': 0.01, 'solver': 'lbfgs'}	0.85	0.86	0.73	0.97	0.36	0.91	0.49	0.61	0.88

0.50	{0: 1, 1: 5}	{'C': 0.01, 'solver': 'lbfgs'}	0.82	0.82	0.80	0.97	0.32	0.89	0.46	0.62	0.88
0.50	{0: 1, 1: 10}	{'C': 0.01, 'solver': 'lbfgs'}	0.71	0.69	0.89	0.98	0.24	0.81	0.37	0.57	0.88
0.60	{0: 1, 1: 1}	{'C': 100, 'solver': 'liblinear'}	0.91	0.98	0.28	0.93	0.59	0.95	0.38	0.32	0.88
0.60	{0: 1, 1: 2}	{'C': 100, 'solver': 'lbfgs'}	0.91	0.96	0.44	0.94	0.52	0.95	0.48	0.46	0.89
0.60	{0: 1, 1: 2.5}	{'C': 0.01, 'solver': 'lbfgs'}	0.87	0.89	0.69	0.96	0.41	0.93	0.51	0.61	0.88
0.60	{0: 1, 1: 3}	{'C': 0.01, 'solver': 'lbfgs'}	0.86	0.88	0.72	0.97	0.39	0.92	0.50	0.61	0.88
0.60	{0: 1, 1: 3.5}	{'C': 0.01, 'solver': 'lbfgs'}	0.85	0.86	0.77	0.97	0.38	0.91	0.51	0.64	0.88
0.60	{0: 1, 1: 5}	{'C': 0.01, 'solver': 'lbfgs'}	0.79	0.79	0.81	0.98	0.30	0.87	0.44	0.60	0.88
0.60	{0: 1, 1: 10}	{'C': 0.01, 'solver': 'liblinear'}	0.67	0.64	0.91	0.99	0.22	0.78	0.35	0.55	0.88
0.68	{0: 1, 1: 1}	{'C': 100, 'solver': 'lbfgs'}	0.91	0.98	0.25	0.92	0.57	0.95	0.35	0.28	0.88
0.68	{0: 1, 1: 2}	{'C': 100, 'solver': 'liblinear'}	0.90	0.95	0.42	0.94	0.50	0.95	0.45	0.43	0.88
0.68	{0: 1, 1: 2.5}	{'C': 10, 'solver': 'lbfgs'}	0.90	0.94	0.48	0.94	0.47	0.94	0.48	0.48	0.89
0.68	{0: 1, 1: 3}	{'C': 1, 'solver': 'lbfgs'}	0.89	0.93	0.53	0.95	0.45	0.94	0.49	0.51	0.88
0.68	{0: 1, 1: 3.5}	{'C': 0.01, 'solver': 'lbfgs'}	0.84	0.85	0.73	0.97	0.35	0.91	0.47	0.60	0.88
0.68	{0: 1, 1: 5}	{'C': 0.01, 'solver': 'lbfgs'}	0.79	0.79	0.79	0.97	0.29	0.87	0.42	0.59	0.88
0.68	{0: 1, 1: 10}	{'C': 0.01, 'solver': 'lbfgs'}	0.70	0.68	0.89	0.98	0.23	0.80	0.37	0.56	0.88
0.70	{0: 1, 1: 1}	{'C': 100, 'solver': 'liblinear'}	0.91	0.98	0.26	0.92	0.58	0.95	0.36	0.29	0.88
0.70	{0: 1, 1: 2}	{'C': 100, 'solver': 'liblinear'}	0.90	0.95	0.41	0.94	0.49	0.94	0.45	0.42	0.88
0.70	{0: 1, 1: 2.5}	{'C': 10, 'solver': 'lbfgs'}	0.90	0.94	0.49	0.94	0.49	0.94	0.49	0.49	0.89
0.70	{0: 1, 1: 3}	{'C': 10, 'solver': 'lbfgs'}	0.90	0.93	0.55	0.95	0.47	0.94	0.51	0.53	0.89
0.70	{0: 1, 1: 3.5}	{'C': 100, 'solver': 'lbfgs'}	0.88	0.92	0.53	0.95	0.41	0.93	0.46	0.50	0.87
0.70	{0: 1, 1: 5}	{'C': 0.01, 'solver': 'lbfgs'}	0.79	0.79	0.81	0.97	0.30	0.87	0.43	0.60	0.88
0.70	{0: 1, 1: 10}	{'C': 0.01, 'solver': 'lbfgs'}	0.70	0.67	0.89	0.98	0.23	0.80	0.36	0.56	0.88
0.75	{0: 1, 1: 1}	{'C': 100, 'solver': 'lbfgs'}	0.91	0.98	0.28	0.93	0.57	0.95	0.38	0.31	0.89
0.75	{0: 1, 1: 2}	{'C': 100, 'solver': 'lbfgs'}	0.91	0.96	0.44	0.94	0.52	0.95	0.48	0.46	0.89
0.75	{0: 1, 1: 2.5}	{'C': 100, 'solver': 'lbfgs'}	0.90	0.94	0.52	0.95	0.49	0.94	0.51	0.52	0.88
0.75	{0: 1, 1: 3}	{'C': 10, 'solver': 'lbfgs'}	0.90	0.93	0.55	0.95	0.47	0.94	0.51	0.53	0.88
0.75	{0: 1, 1: 3.5}	{'C': 100, 'solver': 'lbfgs'}	0.88	0.92	0.59	0.95	0.43	0.93	0.50	0.55	0.88
0.75	{0: 1, 1: 5}	{'C': 0.01, 'solver': 'lbfgs'}	0.79	0.79	0.80	0.97	0.29	0.87	0.43	0.59	0.88
0.75	{0: 1, 1: 10}	{'C': 0.01, 'solver': 'lbfgs'}	0.69	0.67	0.89	0.98	0.23	0.80	0.36	0.56	0.88
0.90	{0: 1, 1: 1}	{'C': 10, 'solver': 'lbfgs'}	0.91	0.98	0.29	0.93	0.60	0.95	0.39	0.32	0.89
0.90	{0: 1, 1: 2}	{'C': 100, 'solver': 'lbfgs'}	0.90	0.95	0.46	0.94	0.50	0.95	0.48	0.47	0.88
0.90	{0: 1, 1: 2.5}	{'C': 100, 'solver': 'lbfgs'}	0.90	0.95	0.50	0.95	0.50	0.95	0.50	0.50	0.88
0.90	{0: 1, 1: 3}	{'C': 10, 'solver': 'liblinear'}	0.90	0.94	0.52	0.95	0.47	0.94	0.50	0.51	0.88

0.90	{0: 1, 1: 3.5}	{'C': 100, 'solver': 'liblinear'}	0.89	0.92	0.59	0.95	0.45	0.94	0.51	0.55	0.88
0.90	{0: 1, 1: 5}	{'C': 100, 'solver': 'lbfgs'}	0.86	0.88	0.68	0.96	0.39	0.92	0.49	0.59	0.88
0.90	{0: 1, 1: 10}	{'C': 0.01, 'solver': 'lbfgs'}	0.69	0.67	0.90	0.98	0.23	0.80	0.36	0.56	0.88
1.00	{0: 1, 1: 1}	{'C': 100, 'solver': 'liblinear'}	0.91	0.98	0.27	0.92	0.58	0.95	0.36	0.30	0.88
1.00	{0: 1, 1: 2}	{'C': 10, 'solver': 'lbfgs'}	0.90	0.95	0.43	0.94	0.50	0.95	0.46	0.44	0.88
1.00	{0: 1, 1: 2.5}	{'C': 100, 'solver': 'lbfgs'}	0.90	0.95	0.47	0.94	0.49	0.94	0.48	0.47	0.88
1.00	{0: 1, 1: 3}	{'C': 100, 'solver': 'lbfgs'}	0.90	0.93	0.55	0.95	0.47	0.94	0.51	0.53	0.88
1.00	{0: 1, 1: 3.5}	{'C': 100, 'solver': 'lbfgs'}	0.88	0.92	0.56	0.95	0.43	0.93	0.49	0.53	0.88
1.00	{0: 1, 1: 5}	{'C': 100, 'solver': 'lbfgs'}	0.86	0.88	0.66	0.96	0.38	0.92	0.48	0.58	0.88
1.00	{0: 1, 1: 10}	{'C': 100, 'solver': 'liblinear'}	0.78	0.77	0.82	0.98	0.28	0.86	0.42	0.59	0.87

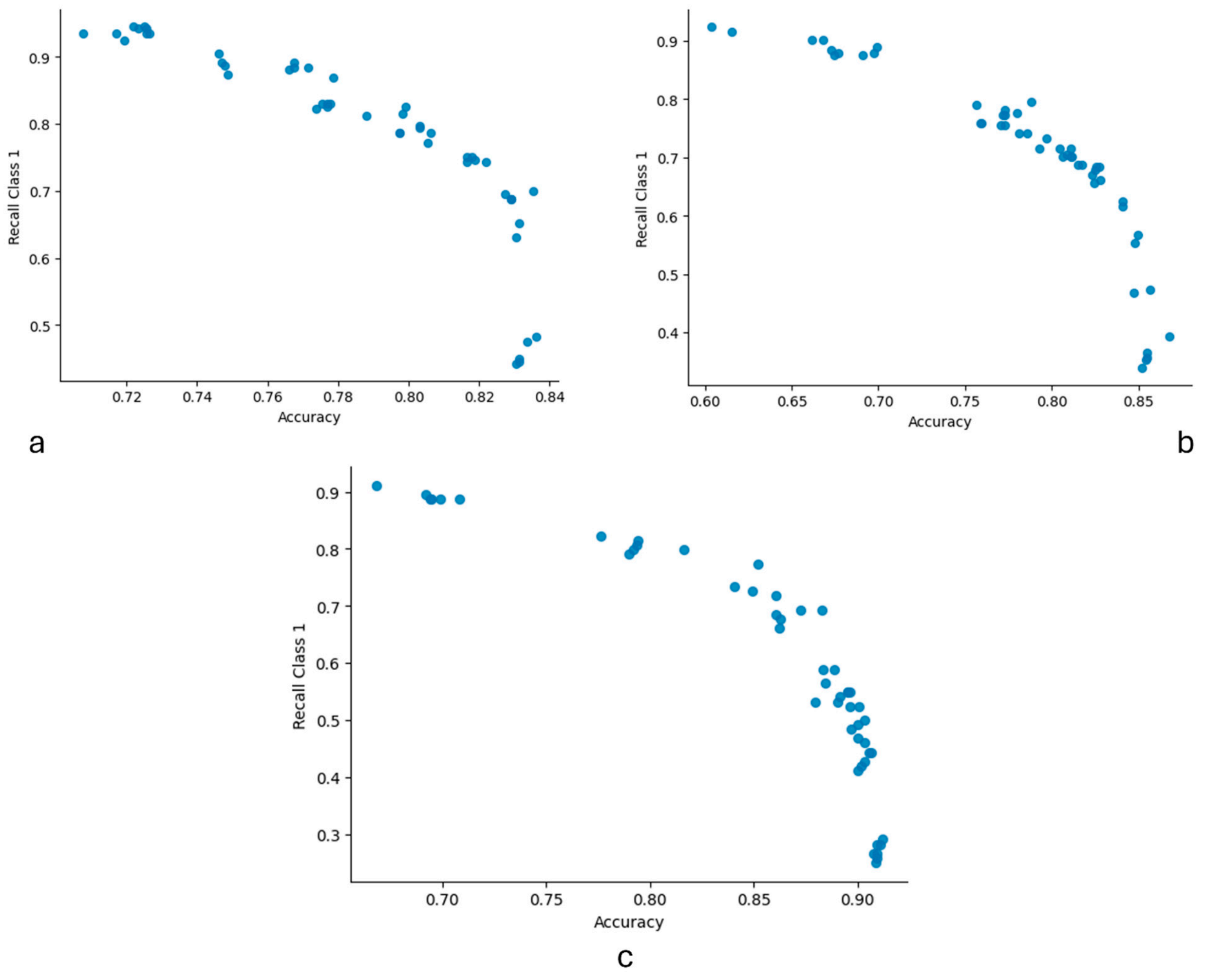

Figure S13: Scatter plots representing the trade-off between accuracy and recall for Class 1 across different models for (a) suicide ideation, (b) suicide planning, and (c) suicide attempt

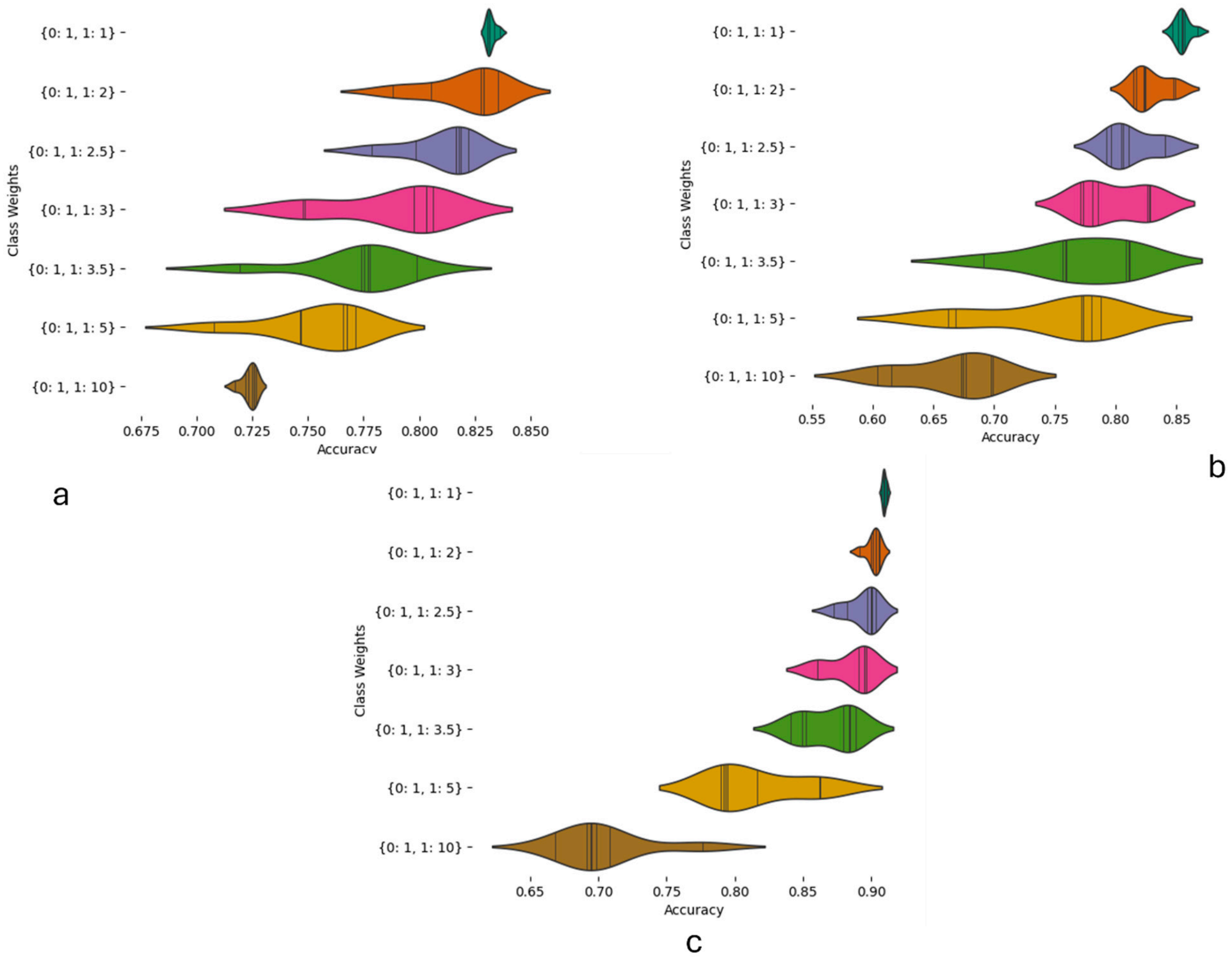

Figure S14: Violin plots representing the distribution of model accuracy across different class weights for (a) suicide ideation, (b) suicide planning, and (c) suicide attempt

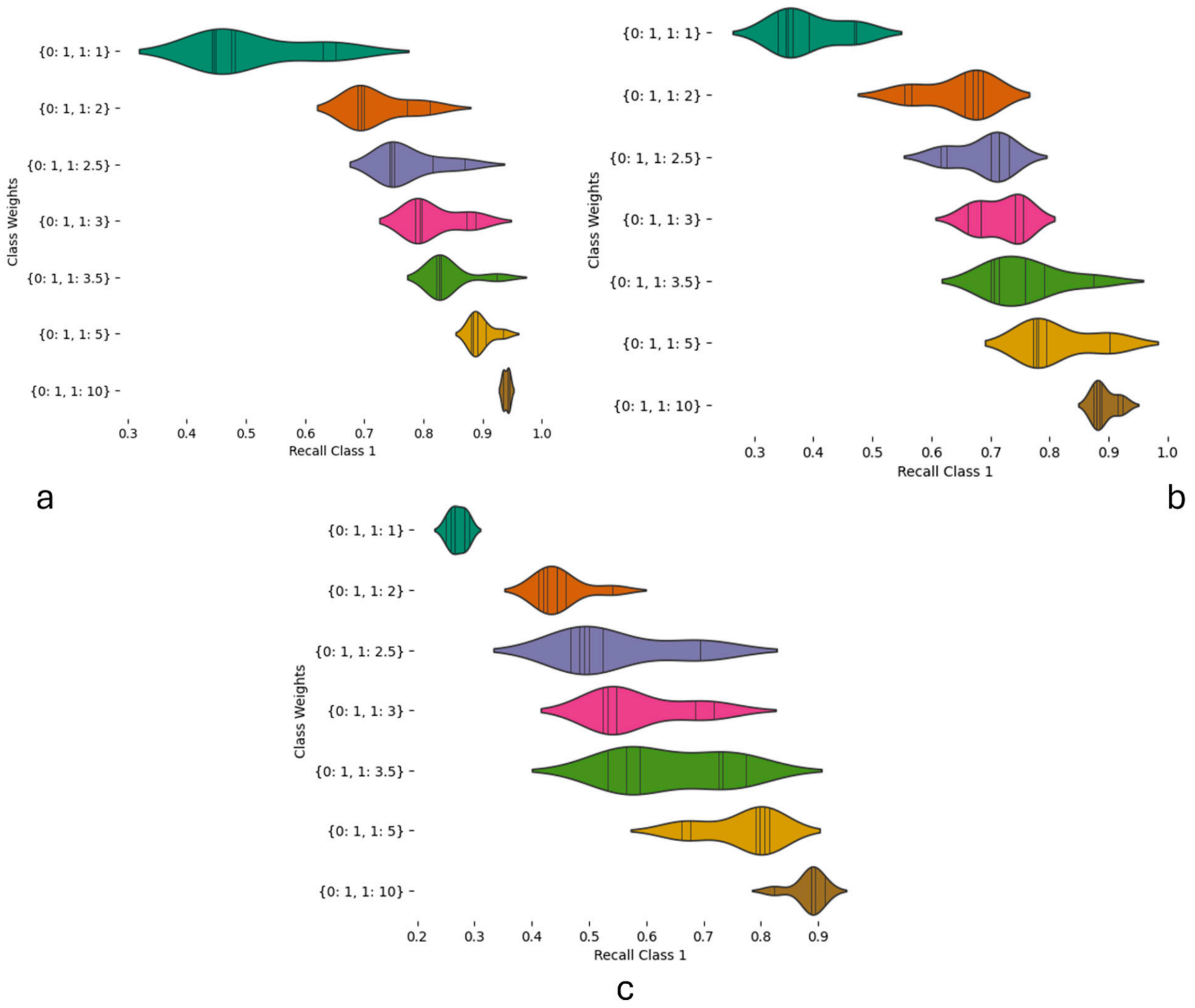

Figure S15: Violin plots representing the distribution of recall class 1 across different class weights for (a) suicide ideation, (b) suicide planning, and (c) suicide attempt
